# Whole genome sequencing in the search for genes associated with the control of SIV infection in the Mauritian macaque model

**DOI:** 10.1038/s41598-018-25071-x

**Published:** 2018-05-08

**Authors:** Marc de Manuel, Takashi Shiina, Shingo Suzuki, Nathalie Dereuddre-Bosquet, Henri-Jean Garchon, Masayuki Tanaka, Nicolas Congy-Jolivet, Alice Aarnink, Roger Le Grand, Tomas Marques-Bonet, Antoine Blancher

**Affiliations:** 1 Institute of Evolutionary Biology, UPF-CSIC, PRBB, Dr. Aiguader 88, 08003 Barcelona, Spain; 2 Catalan Institution of Research and Advanced Studies, ICREA, Passeig de Lluís Companys, 23, 08010 Barcelona, Spain; 3 CNAG-CRG, Centre for Genomic Regulation, CRG, Barcelona Institute of Science and Technology (BIST, Baldiri i Reixac 4, 08028 Barcelona, Spain; 4 Department of Molecular Life Science, Division of Basic Medical Science and Molecular Medicine, Tokai University School of Medicine, Isehara, Kanagawa Japan; 5 CEA – Université Paris-Sud 11 – INSERM U1184, Immunology of Viral Infections and Autoimmune Diseases, IDMIT Department, IBFJ, 92265 Fontenay-aux-Roses, France; 6 Inserm U1173, Simone Veil School of Health Sciences, University of Versailles Saint-Quentin-en-Yvelines, Montigny-le-Bretonneux, France; 7 Genetics Division, Ambroise Paré Hospital (AP-HP), Boulogne-Billancourt, France; 8 Support Center for Medical Research and Education, Tokai University, Isehara, Kanagawa Japan; 9 Laboratoire d’immunogénétique moléculaire (LIMT, EA 3034, Faculté de médecine Purpan, Université Toulouse 3 (Université Paul Sabatier, UPS), Toulouse, France; 10 Laboratoire d’immunologie, CHU de Toulouse, France

## Abstract

In the Mauritian macaque experimentally inoculated with SIV, gene polymorphisms potentially associated with the plasma virus load at a set point, approximately 100 days post inoculation, were investigated. Among the 42 animals inoculated with 50 AID_50_ of the same strain of SIV, none of which received any preventive or curative treatment, nine individuals were selected: three with a plasma virus load (PVL) among the lowest, three with intermediate PVL values and three among the highest PVL values. The complete genomes of these nine animals were then analyzed. Initially, attention was focused on variants with a potential functional impact on protein encoding genes (non-synonymous SNPs (NS-SNPs) and splicing variants). Thus, 424 NS-SNPs possibly associated with PVL were detected. The 424 candidates SNPs were genotyped in these 42 SIV experimentally infected animals (including the nine animals subjected to whole genome sequencing). The genes containing variants most probably associated with PVL at a set time point are analyzed herein.

## Introduction

Experimental infection of macaques with simian immunodeficiency virus (SIV) is an animal model of the HIV infection in humans currently used in numerous laboratories to test new therapies and to elucidate the pathophysiology of the acquired virus-induced immunodeficiency syndrome (AIDS). Among the various macaque species which have been investigated, cynomolgus macaques have been widely used because of the availability of animals bred in captivity. The cynomolgus macaque (*Macaca fascicularis, Mafa*) is native to Southeast Asia where it is widely distributed. Genetic studies based on whole genome sequencing revealed a large genetic variability of natural Asian populations and a strong inter-regional differentiation^1,2^. However, in Mauritius, far from Southeast Asia, a cynomolgus macaque population settled and expanded after few animals were abandoned on the island, five hundred years ago, by sailors who had captured them in Indonesia (Java and/or Sumatra) and Malaysia. As a consequence of the strong bottleneck and complete isolation of the population from the time of its foundation, the polymorphism of the Mauritian macaque population is significantly reduced compared to natural populations^3,4^. The Mauritius cynomolgus macaque population shows about 20% less genetic diversity than the Indonesian–Malaysian population^5^. Although being reduced when compared to Asian macaque populations, the level of heterozygosity in the Mauritian macaque population (2.8 × 10^−3^) was estimated higher than that of humans (~1.0 × 10^−4^)^3^.

A study of the Mauritian macaque model, revealed that a small percentage of SIV-infected macaques control virus replication without antiretroviral treatment^6^. In the Mauritian macaque model, an association between MHC genotype and the control of SIV infection was reported^6–10^. However, the MHC haplotypes associated with control of SIV infection varied from one study to another. We have previously shown that by taking into account multiple comparisons, the MHC haplotype M2 was the sole MHC haplotype to be significantly associated with low set point PVL, and that MHC genotype accounted for only 35% of the inter-individual variability of the set point PVL^6,11^. Thus 65% of the inter-individual set point PVL variability could be partly related to environmental factors or could result from the influence of polymorphic loci located outside the MHC. The aim of the present study is to identify such loci.

In a previous study, Ericsen and col. identified candidate control-modifying loci by sequencing the genomes of 12 SIV-infected Mauritian cynomolgus macaques that exhibited differing viral load at the set point^12^. The 12 animals selected in the study shared a major histocompatibility complex haplotype (referred to as M1 haplotype), which has been found to be enriched in macaques that control SIV^13^.

**Figure 1:**
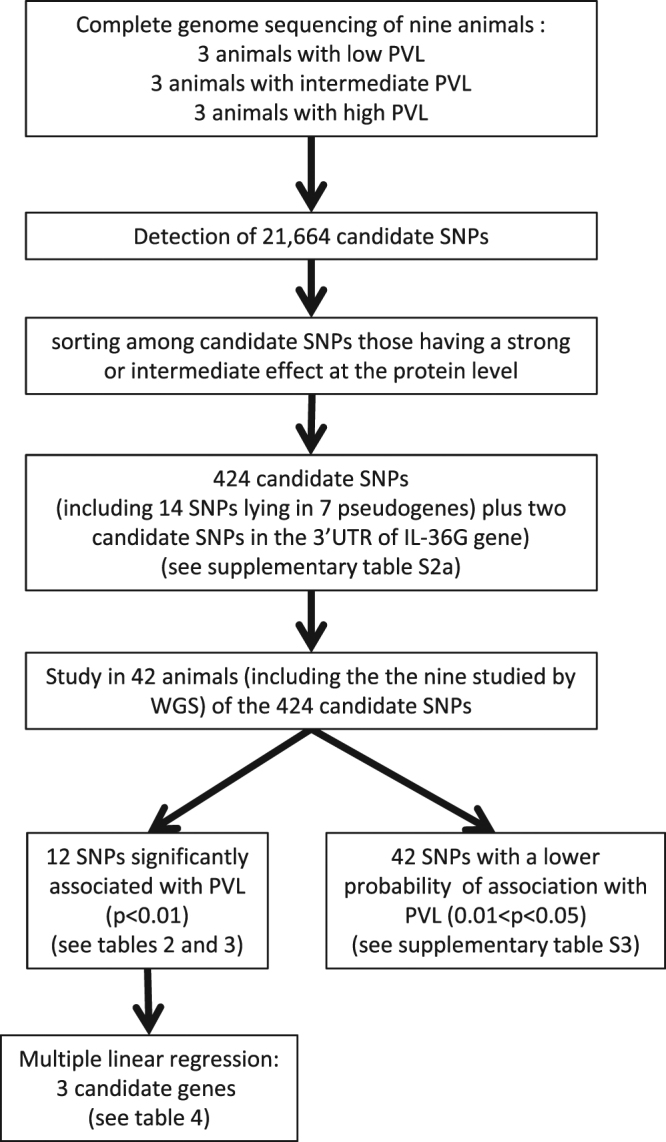
Strategy of gene sequencing used in this study to identify candidate SNPs potentially associated with PVL.

In the present study, we adopted a different strategy that does not take into account the MHC genotypes of the animals and uses a larger panel of individuals. In this multi-phased approach, we first sequenced the genome of nine animals selected from a group of 42 animals used as controls in various experimental protocols (all animals received 50 AID_50_ of the same strain of SIV, and were SIV-naive prior to experimental challenge and did not receive any preventive or curative treatment). The nine individuals were selected as a function of their SIV plasma virus load at the set point (around 100 days post inoculation): three had a plasma virus load (PVL) among the lowest (below the 25th percentile), three had intermediate PVL values (between the 25th and the 75th percentiles) and three had PVL among the highest (above the 75th percentile). The nine complete genomes were used for variant calling. In this first step, attention was focused on variants with a potential functional impact on protein encoding genes (non-synonymous SNPs (NS-SNPs) and splicing variants). A schematic workflow of the study is shown in Fig. 1.

By studying genome-wide data of the nine animals, we detected 424 NS-SNPs possibly associated with PVL. We further genotyped these 424 candidate SNPs in the 42 Mauritian cynomolgus macaques experimentally infected with SIV (including the nine animals subjected to whole genome sequencing). For that purpose, we elaborated a customized sequence capture system. The sequences surrounding the SNPs of interest were captured from the 42 animal genomic DNAs and sequenced by a multiplexed approach by using the Illumina technique.

The genes containing the variants most probably associated with PVL were analyzed.

## Results

### Whole genome sequencing of nine animals

The whole genome of nine Mauritian cynomolgus macaques were sequenced to an average of 20-fold coverage (see Table 1 for details). As previously reported, the Mauritian cynomolgus macaques were genetically closer to a representative of the Malaysian population than to a representative of the Indochinese population^5^. Despite the extreme bottleneck experienced by the Mauritian macaque population^14,15^, the overall level of nucleotide diversity was only 23% smaller than that of the Malaysian cynomolgus macaques^5^. Assuming a single bottleneck (due to the founding effect) followed by an exponential population growth, the number of founders was estimated to be approximately 20 individuals^5^.

**Table 1:** General results of whole genome sequencing of nine animals.

Animal ID number	Draft read numbers^(1)^	Depth^(2)^	Total SNV	Heterozygous SNV	Homozygous SNV	Heterozygosity
9204	581,033,872	20.6	9,760,768	5,959,394	3,801,374	0.00236
11245	765,801,502	22.6	9,7086,27	5,833,119	3,875,508	0.00231
9413	562,065,757	20.1	9,787,656	6,047,099	3,740,557	0.00239
8249	548,219,597	19.6	9,810,492	6,065,823	3,744,669	0.0024
9859	585,458,964	21.0	9,732,886	5,882,941	3,849,945	0.00233
8141	584,297,512	21.0	9,782,593	5,936,862	3,845,731	0.00235
8102	595,837,161	21.5	9,685,199	5,784,255	3,900,944	0.00229
10435	777,622,100	23.7	9,698,080	5,817,415	3,880,665	0.0023
10465	763,698,789	23.1	9,637,930	5,585,852	4,052,078	0.00221

^(1)^Average read length 100 bp.

^(2)^Reads mapped on autosomes.

### Association between variants and PVL

The study of genomic variants in the nine animals selected among the 42 animals with known PVL values at the set point (three with the highest, intermediate or the lowest PVL), led to the characterization of 21,664 SNPs potentially associated with the PVL.

In order to reduce the number of candidate SNPs, we decided to focus our attention on SNPs for which the variations had high or moderate effect on the encoded proteins (non-synonymous SNP (NS-SNPs), variants in splicing regions, nonsense mutations etc.). By applying this filter, we found 424 SNPs potentially implicated (the complete list is given in Supplementary Table S2a, chromosomal locations are summarized in Supplementary Table S2b and genes implicated in HIV infection are listed in Table S2c). Most of these SNPs were inside functional genes (408 SNPs located in 252 functional genes). However, 14 SNPs were located in seven pseudogenes. The list of functional genes was compared to the list of genes involved in HIV infection and replication (http://www.ncbi.nlm.nih.gov/genome/viruses/retroviruses/hiv-1/interactions). Among the 252 genes, 11 correspond to genes the knockdown of which was reported to enhance the HIV replication in various *ex vivo* cellular models (Supplementary Table S2c). The proteins encoded by 25 other genes have been reported to interact with HIV1 proteins and five other genes have been reported in the literature to be related to HIV-1 infection. Finally, 19 other genes encoded proteins involved in the immune responses out of which eight genes were located in the MHC class-II region (Supplementary Table S2d).

Additionally, a SNP density Manhattan plot is shown in Supplementary Figure S1b. Following the strategy used by Ericsen and col^12^. variant sites were selected for which the animals of the two groups with extreme PVL values (three animals with the highest and three animals with the lowest PVL values) were homozygous but for which the two groups differed (one group was homozygous for the reference base the other was homozygous for the variant base) we found 4,532 sites dispersed along the genome. The highest density regions (above the 95th percentile) differed from the candidates regions characterized by Ericsen *et al*.^12^. In the high density regions characterized in the present study, 120 coding genes were identified out of which 17 were related with SIV infection or the immune response (see Supplementary Figure S1b for details).

### Further analysis of 424 candidate SNPs in 42 animals

The totality of the 424 candidate SNPs (including those lying in pseudogenes or in genes encoding unidentified proteins) were then studied in a larger panel of 42 animal samples (which included the nine animals studied by whole genome sequencing). The study of the 424 SNPs in 42 animals demonstrated that only 5 non-synonymous (UMODL1, SLC26A8, MYH13, MYH8, LOC102139876 which corresponds to MAGED4) SNPs located in functional genes displayed probabilities of association with the PVL at the set point (Fisher’s exact test) below 1 × 10^−2^ (see Table 2 for details). Of the 424 SNPs studied in the 42 animals, 69 gave genotypes homologous to those observed for at least one other SNP. The Bonferroni corrected significance threshold for multiple comparisons (355 original markers in this case) was 1.41 × 10^−4^. Therefore, none of the 424 SNPs were significantly associated with PVL after correcting for multiple testing. Other variants in functional genes that show weaker association with PVL scores (0.01 < P < 0.05 by means of Fisher’s exact test) in the study of 42 SIV-infected cynomolgus macaques are listed in the Supplementary Table S3 but will not be discussed further.

**Table 2:** Detection of 5 variants that showed strong association with PVL scores (P < 0.01 by means of exact Fisher’s test) in 42 SIV-infected cynomolgus macaques.

Gene	Chr.	Location of SNP on chromosome*	P-value by exact test	Odds ratio	P-value	95% Cl	interaction with SIV
UMODL1 Uromodulin-Like 1	3	4,684,432	2.4 × 10^−3^	5.9	1.2 × 10^−3^	1.376–29.542	see note “$“
SLC26A8 Solute Carrier Family 26 (Anion Exchanger), Member 8	4	134,811,277	4.3 × 10^−3^	>8.9	9.1 × 10^−3^	1.583 - Inf	most probably none
MYH13 myosin 13	16	10,498,978	5.4 × 10^−3^	4.9	2.6 × 10^−2^	1.129–24.526	most probably none
MYH8 myosin 8	16	10,573,814	5.4 × 10^−3^	4.9	2.6 × 10^−2^	1.129–24.526	most probably none
LOC102139876 melanoma-associated antigen D4	X	50,696,215	7.9 × 10^−3^	7.5	7.9 × 10^−3^	1.468–53.826	most probably none

*The positions are based on the assembly of the Macaca fascicularis genome MacFas5.0 (GCA 000364345.1).﻿

$Uromodulin-Like 1 (olfactorin): Knockdown of uromodulin-like 1 (UMODL1) by siRNA inhibits the early stages of HIV-1 replication in 293T cells infected with VSV-G pseudotyped HIV-1.

As for the interleukin 36 gamma (IL-36G) gene, the study of nine complete genomes revealed a potential association with two NS-SNPs. Because knockdown of IL-36G by siRNA enhances the early stages of HIV-1 replication in an *in vitro* model of epithelial cell infection^16^, the IL-36G gene appeared as one of the most plausible candidate gene. Therefore, we decided to further study the polymorphism of this gene in the macaque model. Looking for other variants inside the IL-36G gene, we detected two SNPs in the 3’UTR of the IL-36G gene which were potentially associated with PVL. We genotyped the 42 animals for the four IL-36G candidate SNPs (two NS and two located in the 3’UTR region). While the study of 42 animals did not confirm a significant association for the two IL-36G NS-SNPs, we found that the two IL-36G 3′UTR SNPs could be associated with PVL (see Table 3 for details). One of the two 3′UTR SNPs was significantly associated with PVL as confirmed by multiple linear regression (see Table 4).

**Table 3:** Good candidates located inside pseudogenes or 3′UTR (IL-36G gene).

Gene	Chr.	Location on chromosome^(1)^	P-value by exact test	Odds ratio	P-value	95% Cl
LOC102130113^(2)^ protein theta pseudogene	5	163,236,012	2.0 × 10^−4^	16.1	3.1 × 10^−3^	1.832–784.237
	5	163,262,889	2.0 × 10^−4^	16.1	3.1 × 10^−3^	1.832–784.237
	5	163,263,047	2.0 × 10^−4^	16.1	3.1 × 10^−3^	1.832–784.237
	5	163,263,341	2.0 × 10^−4^	16.1	3.1 × 10^−3^	1.832–784.237
LOC102128847 beta-citrylglutamate synthase B pseudogene	14	77,876,668	2.4 × 10^−3^	9.9	8.4 × 10^−4^	2.116–59.352
IL36G 3′UTR	13	17,133,365	4.4 × 10^−3^	8.9	3.2 × 10^−3^	1.773–63.019
	13	17,133,758	3.2 × 10^−3^	11.4	2.0 × 10^−3^	1.928–127.17

^(1)^The positions are based on the assembly of the *Macaca fascicularis* genome MacFas5.0 (GCA 000364345.1).

^(2)^The genotypes of the 42 animals were strictly homologous (base of reference/variant) for the four candidate SNPs inside LOC102130113. Therefore, the p values and odds ratio are identical.

**Table 4:** Multiple linear regression.

Marker/gene	Chr.	Location on chromosome^(1)^	P-value
**M2**	4	MHC class-IB region	1.54 × 10^−5^
**M6**	4	MHC class-IB region	3.63 × 10^−6^
**LOC102130113**^(2)^ protein theta pseudogene	5	163,236,012; 163,262,889; 163,263,047; 163,263,341	2.35 × 10^−4^
**IL-36G (3′UTR)**	13	17,133,365	3.91 × 10^−2^
**LOC102139876** (MAGED4)^(3)^	X	50,696,215	1.27 × 10^−3^

^(1)^locations on chromosomes (*Macaca fascicularis* RefSeq assembly GCF_000364345.1).

^(2)^In the LOC102130113 we identified four candidate SNPs for which the 42 animal had identical genotypes. We take into account only one out of the four SNPs in the multiple linear regression model.

^(3)^MAGED4: melanoma-associated antigen D4.

Among variants located in pseudogenes, we found that the probabilities of association for the SNPs located in LOC102130113 (p = 2 × 10^−4^) which corresponds to the “protein theta pseudogene” are close to the threshold of significance (1.41 × 10^−4^). The probability of association of SNP in LOC102128847 which corresponds to the “beta-citrylglutamate synthase B pseudogene” was much lower (see the discussion for more details).

### Multiple linear regression model

We tested the combined impact of the SNPs lying in the 9 good candidate genes (SNPs with probabilities of association <0.001 see Tables 2 and 3) and 7 MHC class IB haplotypes on the control of PVL with a multiple linear regression (MLR) model. We selected the best model by stepwise regression. Both forward and backward stepwise regression yielded the same model with 5 significant predictors, including M2 and M6 MHC haplotypes, LOC102130113, IL-36G 3′UTR and MAGED4, see Table 4 for details). These five markers together explained 67% of the logPVL variance (p = 8.1 × 10^−9^).

## Discussion

We followed a strategy based on the identification of variants with high or moderate predicted effects on encoded proteins and segregating in either controllers or progressors using whole-genome sequences of nine animals. Identified variants were submitted to a posterior validation by targeted genotyping in a larger cohort of 42 individuals. This procedure is not able to identify regulatory regions playing a role in SIV progression, but focuses on non-synonymous SNPs that are readily genotyped in a large cohort of 42 individuals and can be easily interpreted under a gene driven hypothesis. Previously, Ericsen *et al*. scanned whole-genome sequences looking for differentiated linkage disequilibrium blocks^12^. Applying the same whole-genome scanning method, we identified regions differing from those reported by Ericsen *et al*. (see Supplementary Figure S1b). We hypothesize that these differences stem from: (i) the low sample sizes in both studies (ii) the different criteria for selection of the animals for the detection of associated variants and (iii) potential cryptic relatedness among the animals selected for characterization of variants associated with control of the SIV infection. The relatedness of animals is a consequence of the founder effect and isolation of Mauritian macaque population as evoked in the introduction (for more details see Supplementary note S1).

The study of nine complete genome sequences led us to detect 424 candidate SNPs potentially associated with the PVL, out of which 297 were NS variants located in 188 functional genes. The list of these 188 macaque genes in which associated variants were detected, was compared with the list of genes which have been reported in the literature to be associated with HIV1 infection or replication. Among the 188 macaque genes 26 genes are present in the NCBI database. The list of these genes is given in Supplementary Table S2.

A high number of SNPs associated with PVL were found in the class II region of the MHC on macaque chromosome (chr.) 4. Among these SNPs, only one lay in the macaque C4H6orf1 gene homologous to the human C6orf1 gene which is known to be associated in humans with the replication of the HIV (by reference to the NCBI database). Interestingly, it has been reported that knockdown of C6orf1 (chr.6 open reading frame 1) by siRNA inhibits HIV-1 replication in HeLa-derived TZM-bl cells. The genotype of 42 animals did not confirm the association of the all SNPs lying in the MHC class II region. Outside the MHC, probabilities less than 0.01 (Fisher’s exact test) were confirmed for only 5 NS-SNPs which were submitted to MLR analysis. In case of NS-SNPs we detected inside the MYH13 (Myosin, Heavy Chain 13) and MYH8 (Myosin, Heavy Chain 8) genes, it has been reported that HIV-1 protease cleaves the human myosin heavy chains 8 and 13 *in vitro*. However, these two proteins are expressed in skeletal muscles and it is unlikely that they are involved in the fight against SIV. The myosin genes expressed in lymphocytes (MYO1C, MYO1G, and MMYH9) are respectively on chr.16, 3 and 10. The MYO1C gene (*Mafa* chr.16: 1336494–1367073) is very far from MYH13 and MYH8 SNPs (*Mafa* chr.16: 10498978 and 10573814, respectively). In the 2 megabases region around MYH13 and MYH8 we found only three genes which could be involved in the fight against SIV: i) Synthaxin 8, a vesicle trafficking protein that functions in the early secretory pathway, ii) USP43 (Ubiquitin-Specific-Processing Protease 43) which negatively regulates type 1 IFN signaling by downregulating the JAK–STAT pathway, iii) ADPRM (ADP-Ribose/CDP-Alcohol Diphosphatase, Manganese Dependent) knockdown of which by shRNA inhibits HIV-1 replication in cultured Jurkat T-cells (PMID: 19460752). Further study of the polymorphism of these genes is required since the initial screening of association of the nine complete animal genomes could have failed to detect association between these genes and PVL. On the other hand, due to the high number of SNPs studied in 42 animals, it has to be noted that several low probabilities of association could be random. We also characterized a NS-SNP associated with PVL in the UMODL1 (Uromodulin-Like 1) gene. It is important to note that knockdown of UMODL1 by siRNA inhibits the early stages of HIV-1 replication in 293T cells infected with VSV-G pseudotyped HIV-1^17^. However, UMODL1 gene is expressed in urothelial cells and it is highly improbable that this gene is capable of influencing the capacity to resist SIV infection.

As for the NS-SNP in LOC102139876 (melanoma-associated antigen D4, MAGED4), and the one inside the SLC26A8 gene (Solute Carrier Family 26 Anion Exchanger, Member 8), there is no obvious argument in favor of the role of these two genes in the control of SIV infection. Even though MAGED4 encoded protein has been suggested to enhance ubiquitin ligase activity of RING-type zinc finger-containing E3 ubiquitin-protein ligases^18^, its role in the control of SIV infection remains to be demonstrated. Exploration of the macaque genome in the vicinity of these two genes did not reveal any other plausible candidate gene.

Regarding the SNPs located in pseudogenes, the mechanism of their potential role in the control of SIV infection is not obvious. For that reason, the genome map of *M. fascicularis* around these two pseudogenes was investigated in the search for genes which could play a role in the fight against the SIV.

The SNP at position chr.14:77876668 is inside *Mafa* RIMKLBP (beta-citrylglutamate synthase B) retropseudogene (LOC102128847). No functional gene involved in the control of SIV infection was found in the vicinity of the SNP. By blast we identified other RIMLK retro-pseudogenes in the *Mafa* genome (chr.17:28585983–28587204 and chr.1:77891494–77892035) and LOC102128847-like sequences associated with *Mafa* or *M. mulatta* (*Mamu)* MHC genes (5 and 45 sequences, respectively, as shown in Supplementary Table S4). In the *Mamu* MHC, the LOC102128847-like sequence was annotated “KIAA1238”^19^. Noteworthy, *Mafa* MCH associated LOC102128847-like sequences have either an “A” or a “G” at the SNP site while in all other RIMKLBP pseudogenes (chr.1, 14, 17) and the *Mafa* functional RIMKLBP exon 2 (chr.11) there is an “A” at the SNP site. It is possible that A/G SNP could correspond to the polymorphism of the LOC102128847-like sequences associated with MHC. A comparison of LOC102128847-like SNP genotypes with MHC genotypes in the class IB region of the 42 animals under study, led to the conclusion that haplotypes M2 and M4 were associated with LOC102128847-like sequences having a “A” (KT331084 and KJ913137, respectively), while MHC haplotypes M3, M6 and M7 are associated with LOC102128847-like sequences having a “G” (KT330809, KJ48959 and KJ48959-NEW, respectively). Finally, MHC haplotypes M1 and M5 have most probably no LOC102128847-like sequences (see Supplementary Figure S2 for details). The odd ratios and *p* values were recalculated for the SNP located inside MHC and it was found that the presence of a “G” (haplotypes M3, M6 and M7) was associated with a low PVL value (p-value = 0,0048, odds ratio = 7,54) while the absence of LOC102128847-like sequences in the MHC (haplotypes M1 and M5) was associated with a high PVL (and p = 0.0044, odds ratio = 8.0). These conclusions are in accordance with the previous results already reported on 45 animals (which include the 42 animals on which are based the present study)^6^. It is of interest that the whole-genome sequence association study based on nine animals revealed numerous candidate SNPs inside genes of the class II MHC region but none in class IB region. These results could be explained by a sampling effect: the MHC genotype of the nine animals chosen for the initial study biased the detection of candidate SNPs in the MHC class II region. This could also be explained by the high number of highly homologous MHC class IB genes (from 10 to 15 per haploid genome in *Mafa*). This redundancy of class IB genes compromises the attribution of short reads to multiple genomic references.

It is interesting to note that the SNP of LOC102130113 (protein theta pseudogene) is within the intron 1 of the MARCH-1 gene which encodes a protein involved in the expression of MHC class-II proteins at the surface of antigen presenting cells. It is possible that these four SNPs of LOC102130113 significantly associated with PVL in the MLR model, are in linkage disequilibrium with SNPs associated with the level of expression of MARCH-1. The MARCH-1 protein controls the expression of MHC class II molecules at the surface of dendritic cells which present the antigenic peptides to the T lymphocytes. Moreover, it was reported that MARCH-1 protein forms heterodimers with MARCH-8 and that the later controls the replication of the HIV^20,21^. The level of expression of MARCH-1 in macaques (before and after SIV inoculation) remains to be determined in order to confirm the potential association between the expression of the MARCH-1 gene (and/or MARCH-1 protein) and the capacity to control the replication of the SIV.

As for the IL-36G gene, the study of nine complete genomes revealed a potential association with two NS-SNPs. The search for other variants inside the IL-36G gene, led to the detection of two SNPs in the 3′UTR of the predicted transcribed region of the IL-36G gene which were potentially associated with PVL. It was decided therefore to genotype the 42 animals for these four IL-36G SNPs (two NS and two in the 3′UTR). The study of 42 animals did not confirm a significant association for the two IL-36G NS-SNPs. By contrast, by MLR, we found that one of the two IL-36G 3′UTR SNPs was significantly associated with PVL (Table 4). Because this SNPs is located in the 3′ UTR of the IL-36G gene, one can hypothesize that it might be associated with the level of expression of the IL-36G messengers.

In conclusion, this study confirms that in the MHC class-IB two MHC haplotypes M2 and M6 are significantly associated with the low PVL at the set point (in the MLR model presented in Table 4, the respective “p” values are 1.54 × 10^−5^ and 3.63 × 10^−6^). Outside MHC, the MLR model suggests significant association with SNPs located in the intron 1 of MARCH-1 gene, in the MAGED4 gene and in the 3′UTR of IL-36G gene. The protein encoded by MARCH-1 is involved in the control of MHC class-II proteins expression at the surface of dendritic cells. Further studies are required to explore the influence of MARCH-1 genotype on the level of expression of MHC class-II protein at the surface of macaque dendritic cells. The IL-36G gene is a plausible candidate gene since knockdown of the gene using siRNA enhances the early stages of HIV-1 replication in HeLa-CD4 cells^16^ and in 293 T cells^17^. The SNP located in the 3′UTR of the IL-36G gene could influence the stability of the IL-36G mRNA and could affect the expression level of the protein.

Further studies are required to confirm the influence of the 3′UTR SNP on the level of expression of IL-36G in macaques. Although the present study is based on a limited number of animals, several plausible candidate genes have been identified and the strong impact of the MHC class-IB region on the control of SIV infection in the Mauritian macaque model has been confirmed.

## Methods

### Ethics statement

The DNA samples used in this study are historical samples derived from previous SIV therapeutic trials and no further samples were collected for the current study^6^.

All experiments were performed in accordance with the relevant guidelines and regulations. Adult cynomolgus macaques (*Macaca fascicularis*) imported from Mauritius were handled in accordance with European guidelines for NHP care (EU Directive N 63/2010). This study was approved and accredited under statement number 12-006 by the Ethical Animal Committee of the CEA “Comité d’Ethique en Expérimentation Animale” registered by the French Research Ministry under number 44. The CEA complies with the Standards for Human Care and Use of Laboratory Animals of the Office for Laboratory Animal Welfare (OLAW) (USA) under OLAW Assurance number A5826-01.

### Animals

Forty-two male cynomolgus monkeys from Mauritius Island (Noveprim, Bioprim) were inoculated with 50 animal infectious dose 50% (50 AID50) of SIVmac251 isolate by the intravenous (IV, N = 29) or the intrarectal (IR, N = 13) route. The animals received no treatment before or after SIV inoculation (for more details see Aarnink *et al*.^6^).

### Determination of plasma viral load

Plasma viral load (PVL) was measured by quantitative RT-PCR as described by Karlsson *et al*.^22^ (for more details see Aarnink *et al*.^6^). PVL approximately 100 days (range 95–128) after SIV inoculation corresponding to the set-point of viremia was used in the present study. As PVL followed a log-normal distribution, we employed the logarithm of PVL in all our calculations.

### DNA extraction and MHC genotyping

Genomic DNA was extracted from peripheral blood using either the QIAamp DNA Blood mini Kit (Qiagen, Courtaboeuf, France) or a standard phenol-chloroform method. The MHC genotype was determined with 20 microsatellites scattered across the MHC region^6^. The MHC DRB haplotypes were determined by DGGE sequencing as previously described by Blancher *et al*.^23^.

### Complete genome sequencing

For six animals, the library construction, sequencing, and initial quality check were performed at Beijing Genomics Institute (Shenzhen, China) (for details see: Osada *et al*.^5^). As for the three remaining animals the library construction, sequencing, and initial quality check was performed at the CNAG using standard Illumina protocols (Barcelona, Spain).

### Calling of variants and search for their functional impact

All raw sequence reads were mapped to the draft genome of the cynomolgus macaque macFas5 reference sequence using BWA-MEM 0.7.5a-r405 with default parameters. Picard tools (https://github.com/broadinstitute/picard) were used to mark and remove PCR duplicates and to aggregate alignments and generate a single BAM file for each individual.

Whole-genome alignments were deposited to the European Nucleotide Archive^19^ under the accession number PRJNA (deposit in databank under submission).

Single nucleotide polymorphisms were called independently for each individual with FreeBayes^24^ using the following parameters:–standard-filters–no-population-priors -p 2–report-genotype-likelihood-max -C 3. A callability mask was constructed to identify regions of the reference genome were variants could not be confidentially called. Segments in the genome covered by less than four reads with a minimum base quality of 20 and mapping quality of 10 were identified in each individual. Any site outside the intersection of all such regions was excluded from downstream analyses. The total number of autosomal bases after this filtering was 2,386,325,518. Next, annotation data was added to the genotypes using SnpEff 4.0e with the *Macaca fascicularis* built-in database in order to obtain a prediction of the effect on the encoded proteins^25^.

In order to identity variants putatively involved in the differential SIV progression between groups, we selected polymorphisms with a high to moderate predicted effect (based on the SnpEff 4.0e annotation) segregating exclusively in one of the extreme viral load groups (low or high). To increase our power to detect such sites, we also allowed observations of the disruptive allele in the intermediate PVL group. This procedure captured 424 variants distributed in 279 genes (Supplementary Table 2B).

### Sequence capture and next generation sequencing

Barcoded-library DNA samples from the 42 animals were prepared with an Ion Xpress Plus Fragment Library Kit and Ion Xpress barcode Adaptors 1–96 Kit according to the manufacture’s protocol for 200 base-read sequencing (Life Technologies/Thermo Fisher Scientific). Five hundred nanograms of the genomic DNAs were used for the preparation of each DNA library. DNA samples were fragmented with a M220 Focused-ultrasonicator (Covaris). Each DNA library was amplified by eight cycles of PCR. The DNA size and quantitation for each library was measured by an Agilent 2100 Expert Bioanalyzer using the Agilent High Sensitivity DNA Kit (Agilent Technologies). Each barcoded-library was pooled at equimolar concentrations then diluted according to the manufacture’s recommendation. Target barcoded-libraries including the variant sites from the pooled barcoded-libraries were enriched according to the manufacture’s protocol of SeqCap EZ Developer (Roche). The probes for the sequence capture method were designed in total 426 target regions that cover 493 variants. Enriched target barcoded-library was sequenced by Ion PGM system (Life Technologies/Thermo Fisher Scientific). Emulsion PCR (emPCR) was performed using the target barcoded-library with the Ion PGM Template OT2 200 Kit on an Ion OneTouch 2 automated system (Life Technologies/Thermo Fisher Scientific). After the emulsion was automatically broken with the OneTouch 2 instrument, the beads carrying the single-stranded DNA templates were enriched according to the manufacture’s recommendation. Sequencing was performed using the Ion PGM Sequencing 200 Kit and Ion 318 Chip Kit v2. The raw data processing and base-calling, trimming and output of quality-filter sequence reads that were binned on the basis of the Ion Xpress Barcodes into 42 separate sequence fastq files, were all performed by the Torrent Suite 4.2.1 software (Life Technologies/Thermo Fisher Scientific) with full processing for shotgun analysis. These files were further quality trimmed to remove poor sequence at the end of the reads with QVs of less than 10. The trimmed and barcode-binned sequence reads were used for automatically genotyping using Torrent Variant Caller (Life Technologies/Thermo Fisher Scientific). Finally, we carefully checked all variant sites by defining five and under read numbers as the not detected (ND) category.

### Calculations of odd ratio, probabilities of association

Animals were sorted as a function of the PVL at the set point and were separated into two groups of 21 animals each (a group with high PVL and the other with low PVL). All allele frequencies in the two groups were compared by Fisher’s exact test and odds ratio with 95% confidence interval. All statistical analyses were performed with the use of R software, version R-3.3.2 (https://cran.r-project.org/index.html). Comparisons of relative frequencies were tested for significance using the chi-square test for 2 × 2 tables. The Bonferroni correction was applied since multiple tests were carried out.

### Multiple linear regression (MLR)

Influence of genetic polymorphisms on logarithm-transformed PVL (logPVL) was tested using a multivariate linear model with the “lm” function in the “R” environment. The SNPs genotypes were treated as follows: 0 when the variant was absent, 1 when the individual was heterozygous, 2 when the animal was homozygous for the variant. Likewise, the 7 MHC genotypes were converted into numerical variables, depending on their absence (0), presence in simple dose (1) or presence in double dose (2). We selected the best model in a stepwise manner (either forward or backward) using the Akaike information criterion. We reported the p value of the model with the significant predictors as well as the ANOVA p values for these predictors and the adjusted R-squared coefficient as the proportion of variance explained.

## Electronic supplementary material

Supplementary tables and figures
