# Supplementary material for: Whole genome sequencing in the search for genes associated with the control of SIV infection in the Mauritian macaque model
Source: Sci Rep. 2018 May 8;8:7131. doi: 10.1038/s41598-018-25071-x (PMC5940699; doi:10.1038/s41598-018-25071-x)
Supplement: Supplementary file 1 — Supplementary tables and figures [file 41598_2018_25071_MOESM1_ESM.docx]

**Title: Whole genome sequencing in the search for genes associated with the control of SIV infection in the Mauritian macaque model**

**Running title: Genes associated with PVL in SIV infected macaques**

Marc de Manuel ^1,2,3^, Takashi Shiina ^4^, Shingo Suzuki ^4^, Nathalie Dereuddre-Bosquet ^5^, Henri-Jean Garchon^6^, Masayuki Tanaka ^7^, Nicolas Congy-Jolivet ^8,9^, Alice Aarnink ^8^, Roger Le Grand ^5^, Tomas Marques-Bonet ^1,2,3^, Antoine Blancher ^8,9^,

^1^ Institute of Evolutionary Biology,UPF-CSIC, PRBB, Dr. Aiguader 88, 08003 Barcelona, Spain.

^2^ Catalan Institution of Research and Advanced Studies, ICREA, Passeig de Lluís Companys, 23, 08010, Barcelona, Spain

^3^ CNAG-CRG, Centre for Genomic Regulation, CRG, Barcelona Institute of Science and Technology (BIST, Baldiri i Reixac 4, 08028 Barcelona, Spain

^4^ Department of Molecular Life Science, Division of Basic Medical Science and Molecular Medicine, Tokai University School of Medicine, Isehara, Kanagawa, Japan,

^5^ CEA – Université Paris-Sud 11 – INSERM U1184, Immunology of Viral Infections and Autoimmune Diseases, IDMIT Department, IBFJ, 92265, Fontenay-aux-Roses, France

^6^ Inserm U1173, Simone Veil School of Health Sciences, University of Versailles Saint-Quentin-en-Yvelines, Montigny-le-Bretonneux, France; Genetics Division, Ambroise Paré Hospital (AP-HP), Boulogne-Billancourt, France

^7^ Support Center for Medical Research and Education, Tokai University, Isehara, Kanagawa, Japan.

^8^ Laboratoire d’immunogénétique moléculaire (LIMT, EA 3034, Faculté de médecine Purpan, Université Toulouse 3 (Université Paul Sabatier, UPS)

^9^ Laboratoire d’immunologie, CHU de Toulouse, France

**Supplementary tables and figures.**

**Supplementary table S1:**  Animals used in this study (N=42).

| animal ID# | animal identity code (1) | Route of inoculation (2) | log PVL value at the set point (3) | MHC haplotype in the class IB region (4) |
| --- | --- | --- | --- | --- |
| 1 | 23037 | IR | 0.00 | M2/M6 |
| 2 | 9204 | IV | 1.78 | M4/M6 |
| 3 | 11137 | IV | 1.78 | M2/M5 |
| 4 | 11245 | IV | 1.78 | M3/M2 |
| 5 | 11637 | IV | 1.78 | M3/M2 |
| 6 | 15885 | IR | 1.96 | M7/M2 |
| 7 | OBHJ6 | IR | 2.15 | M3/M6 |
| 8 | 9413 | IV | 2.22 | M3/M1 |
| 9 | OBG7 | IR | 2.23 | M2/M6 |
| 10 | OBPR6 | IR | 2.28 | M2/M4 |
| 11 | Z776 | IV | 2.48 | M5/M6 |
| 12 | 10515 | IV | 2.53 | M2/rec*** |
| 13 | 10228 | IV | 2.57 | M3/ rec*** |
| 14 | 15461 | IR | 2.61 | M2/M3 |
| 15 | 11296 | IV | 2.77 | M3/M2 |
| 16 | OBFE6 | IR | 2.90 | M1/M2 |
| 17 | 9691 | IV | 2.91 | M3/M3 |
| 18 | 20595 | IV | 2.96 | M2/rec*** |
| 19 | 8249 | IV | 3.00 | M1/M4 |
| 20 | 23060 | IR | 3.09 | M3/M3 |
| 21 | 20351 | IR | 3.09 | M2/M1 |
| 22 | 20654 | IV | 3.14 | M1/M3 |
| 23 | 15232 | IR | 3.27 | M1/M2 |
| 24 | 10116 | IV | 3.40 | M5/M1 |
| 25 | 15693 | IV | 3.43 | M1/M2 |
| 26 | 473 | IV | 3.50 | M1/M4 |
| 27 | 10024 | IV | 3.54 | M3/M3 |
| 28 | 9345 | IV | 3.61 | M4/M1 |
| 29 | 20525 | IV | 3.93 | M2/M4 |
| 30 | 9859 | IV | 4.00 | M1/M1 |
| 31 | 8141 | IV | 4.05 | M1/M2 |
| 32 | 15596 | IV | 4.05 | M1/M5 |
| 33 | 23014 | IR | 4.12 | M4/M4 |
| 34 | OBRG6 | IR | 4.26 | M1/M1 |
| 35 | 4763 | IV | 4.33 | M1/M4 |
| 36 | 8102 | IV | 4.51 | M1/M2 |
| 37 | 11360 | IV | 4.83 | M1/M3 |
| 38 | Z860 | IV | 4.97 | M1/M3 |
| 39 | Z857 | IV | 5.24 | M4/M3 |
| 40 | OBRF6 | IR | 5.27 | M1/M4 |
| 41 | 10435 | IV | 5.40 | M1/M1 |
| 42 | 10465 | IV | 5.56 | M4/rec*** |

**Legend of supplementary table S1.**

1. Nine animals for which high quality DNA was available in large quantities were selected for complete genome sequencing: three (highlighted in blue) with a plasma virus load (PVL) among the lowest (below the 25th percentile), three (highlighted in green) with intermediate PVL values (between the 25th and the 75th percentiles) and three (highlighted in violet) among the highest PVL values (above the 75th percentile).
2. Animals were inoculated intrarectally (IR) or intravenously (IV) with SIVmac251
3. As PVL at the set point (around 100 days after inoculation) followed a log-normal distribution, we employed the logarithm of the PVL in all calculations.
4. Rec : MHC recombinant haplotype

**Supplementary table S2a: Description of 424 SNPs studied in 42 animals**

| **#CHROM** | **localization on chromosome ^(1)^** | **REF ^(2)^** | **ALT ^(3)^** | **GENE** | **EFFECT** | **IMPACT ^(4)^** | **p ^(5)^** |
| --- | --- | --- | --- | --- | --- | --- | --- |
| chr01 | 534,446 | T | G | olfactory receptor 1019 | missense_variant | MODERATE | >0.05 |
| chr01 | 534,689 | T | C | olfactory receptor 1019 | missense_variant | MODERATE | >0.05 |
| chr01 | 1,345,188 | T | A | olfactory receptor 2AK2-like | stop_lost | HIGH | >0.05 |
| chr01 | 15,110,417 | G | A | TARBP1 | missense_variant | MODERATE | >0.05 |
| chr01 | 15,898,215 | C | T | KCNK1 | missense_variant | MODERATE | **3.10 x 10^-02^** |
| chr01 | 53,715,653 | T | C | LOC102131552 | missense_variant | MODERATE | >0.05 |
| chr01 | 90,079,149 | T | C | FCGR2B | missense_variant | MODERATE | >0.05 |
| chr01 | 90,079,172 | A | T | FCGR2B | missense_variant | MODERATE | >0.05 |
| chr01 | 91,720,308 | T | C | SLAMF9 | missense_variant | MODERATE | >0.05 |
| chr01 | 92,754,185 | G | C | PYHIN1 | missense_variant | MODERATE | >0.05 |
| chr01 | 93,054,081 | G | A | SPTA1 | missense_variant | MODERATE | **3.10 x 10^-02^** |
| chr01 | 93,120,108 | G | C | olfactory receptor 10X1 | missense_variant | MODERATE | **3.10 x 10^-02^** |
| chr01 | 93,151,566 | G | A | olfactory receptor 6Y1 | missense_variant | MODERATE | **3.10 x 10^-02^** |
| chr01 | 93,241,271 | T | A | olfactory receptor 10R2 | missense_variant | MODERATE | **3.10 x 10^-02^** |
| chr01 | 93,241,928 | G | T | olfactory receptor 10R2 | missense_variant | MODERATE | **3.10 x 10^-02^** |
| chr01 | 94,889,497 | A | G | SH2D2A | missense_variant | MODERATE | >0.05 |
| chr01 | 133,983,777 | G | C | trafficking protein particle complex subunit 2-like protein | stop_lost | HIGH | >0.05 |
| chr01 | 134,128,753 | A | C | RPL5 | missense_variant | MODERATE | >0.05 |
| chr01 | 135,010,543 | A | G | BRDT | missense_variant | MODERATE | >0.05 |
| chr01 | 153,221,887 | G | A | LRRIQ3 | missense_variant | MODERATE | >0.05 |
| chr01 | 153,238,581 | G | A | LRRIQ3 | missense_variant | MODERATE | >0.05 |
| chr01 | 170,634,143 | A | G | C1H1orf168 | missense_variant | MODERATE | >0.05 |
| chr01 | 196,226,110 | T | C | SPOCD1 | missense_variant | MODERATE | >0.05 |
| chr01 | 204,998,211 | G | C | TCEA3 | missense_variant | MODERATE | >0.05 |
| chr01 | 205,911,978 | T | C | ZBTB40 | missense_variant | MODERATE | >0.05 |
| chr01 | 205,924,683 | C | A | ZBTB40 | missense_variant | MODERATE | >0.05 |
| chr01 | 206,538,299 | G | A | HSPG2 | missense_variant | MODERATE | >0.05 |
| chr01 | 206,571,718 | A | G | HSPG2 | missense_variant | MODERATE | >0.05 |
| chr01 | 206,600,814 | C | T | LDLRAD2 | missense_variant | MODERATE | >0.05 |
| chr01 | 206,603,097 | G | A | LDLRAD2 | missense_variant | MODERATE | >0.05 |
| chr01 | 206,829,700 | C | A | RAP1GAP | missense_variant | MODERATE | >0.05 |
| chr01 | 212,700,961 | G | A | TMEM82 | missense_variant | MODERATE | >0.05 |
| chr01 | 213,068,410 | A | C | not identified | missense_variant | MODERATE | >0.05 |
| chr01 | 213,142,281 | GT | TC | FHAD1 | missense_variant | MODERATE | >0.05 |
| chr02 | 41,161,082 | A | C | PRR23A | missense_variant | MODERATE | >0.05 |
| chr02 | 91,411,251 | A | T | FETUB | missense_variant | MODERATE | **4.30 x 10^-02^** |
| chr02 | 91,428,934 | G | C | HRG | missense_variant | MODERATE | >0.05 |
| chr02 | 91,431,651 | G | T | HRG | missense_variant | MODERATE | >0.05 |
| chr02 | 92,343,568 | C | T | ITGA9 | missense_variant | MODERATE | >0.05 |
| chr02 | 92,375,695 | G | A | ITGA9 | missense_variant | MODERATE | >0.05 |
| chr02 | 92,396,018 | A | G | ITGA9 | missense_variant | MODERATE | >0.05 |
| chr02 | 92,954,416 | A | G | DLEC1 | missense_variant | MODERATE | >0.05 |
| chr02 | 93,137,622 | G | A | SLC22A13 | missense_variant | MODERATE | >0.05 |
| chr02 | 93,139,041 | A | G | SLC22A13 | missense_variant | MODERATE | >0.05 |
| chr02 | 93,218,879 | G | C | XYLB | missense_variant | MODERATE | >0.05 |
| chr02 | 93,276,701 | T | C | XYLB | missense_variant | MODERATE | >0.05 |
| chr03 | 4,684,432 | G | C | UMODL1 | missense_variant | MODERATE | **2.40 x 10^-03^** |
| chr03 | 6,989,766 | T | C | IGSF5 | missense_variant | MODERATE | >0.05 |
| chr03 | 6,989,777 | A | T | IGSF5 | missense_variant | MODERATE | >0.05 |
| chr03 | 18,009,821 | G | A | LTN1 | missense_variant | MODERATE | >0.05 |
| chr03 | 45,261,697 | T | G | MUC3A | missense_variant | MODERATE | >0.05 |
| chr03 | 88,848,526 | G | A | PDE1C | missense_variant | MODERATE | **3.10 x 10^-02^** |
| chr03 | 121,174,620 | A | G | nucleolar protein 56-like | missense_variant | MODERATE | >0.05 |
| chr03 | 121,174,768 | C | T | nucleolar protein 56-like | missense_variant | MODERATE | >0.05 |
| chr03 | 183,555,585 | G | A | AOC1 | missense_variant | MODERATE | >0.05 |
| chr04 | 7,373,248 | T | C | QKI | missense_variant | MODERATE | >0.05 |
| chr04 | 32,621,997 | T | C | polyamine-modulated factor 1 | missense_variant | MODERATE | >0.05 |
| chr04 | 74,417,027 | C | G | COQ3 | missense_variant | MODERATE | >0.05 |
| chr04 | 74,933,782 | C | T | FBXL4 | missense_variant | MODERATE | >0.05 |
| chr04 | 129,342,595 | C | G | NCR2 | missense_variant | MODERATE | >0.05 |
| chr04 | 129,343,435 | G | A | NCR2 | missense_variant | MODERATE | >0.05 |
| chr04 | 130,822,594 | C | T | id196641 | splice_donor_variant | HIGH | >0.05 |
| chr04 | 134,355,196 | C | T | ETV7 | stop_gained | HIGH | >0.05 |
| chr04 | 134,372,461 | C | G | ETV7 | missense_variant | MODERATE | >0.05 |
| chr04 | 134,377,211 | A | G | ETV7 | missense_variant | MODERATE | >0.05 |
| chr04 | 134,413,518 | G | C | C4H6orf222 | missense_variant | MODERATE | >0.05 |
| chr04 | 134,416,334 | C | T | C4H6orf222 | missense_variant | MODERATE | >0.05 |
| chr04 | 134,426,723 | T | C | PNPLA1 | missense_variant | MODERATE | >0.05 |
| chr04 | 134,435,800 | C | T | PNPLA1 | missense_variant | MODERATE | >0.05 |
| chr04 | 134,811,277 | A | G | SLC26A8 | missense_variant | MODERATE | **4.30 x 10^-03^** |
| chr04 | 134,920,750 | C | T | SRPK1 | missense_variant | MODERATE | >0.05 |
| chr04 | 135,915,402 | A | G | UHRF1BP1 | missense_variant | MODERATE | >0.05 |
| chr04 | 135,950,902 | G | C | UHRF1BP1 | missense_variant | MODERATE | >0.05 |
| chr04 | 136,572,167 | C | T | C4H6orf1 | missense_variant | MODERATE | >0.05 |
| chr04 | 137,496,687 | C | A | WDR46 | missense_variant | MODERATE | >0.05 |
| chr04 | 137,583,543 | C | T | HSD17B8 | missense_variant | MODERATE | >0.05 |
| chr04 | 137,601,951 | G | A | COL11A2 | missense_variant | MODERATE | >0.05 |
| chr04 | 137,602,797 | T | G | COL11A2 | missense_variant | MODERATE | >0.05 |
| chr04 | 137,616,016 | A | T | COL11A2 | missense_variant | MODERATE | >0.05 |
| chr04 | 137,621,215 | G | A | COL11A2 | missense_variant | MODERATE | >0.05 |
| chr04 | 137,622,894 | G | A | COL11A2 | missense_variant | MODERATE | >0.05 |
| chr04 | 137,720,007 | G | A | MHC class II antigen, DP beta 1 chain | missense_variant | MODERATE | >0.05 |
| chr04 | 137,728,812 | C | T | MHC class II antigen, DP beta 1 chain | start_lost | HIGH | >0.05 |
| chr04 | 137,731,324 | G | T | MHC class II antigen, DP alpha 1 chain | missense_variant | MODERATE | >0.05 |
| chr04 | 137,878,704 | T | G | MHC class II antigen, DM beta chain | missense_variant | MODERATE | >0.05 |
| chr04 | 137,991,289 | C | A | TAP2 | missense_variant | MODERATE | >0.05 |
| chr04 | 138,026,587 | A | T | MHC class II antigen, DO beta chain | missense_variant | MODERATE | >0.05 |
| chr04 | 138,027,410 | A | G | MHC class II antigen, DO beta chain | missense_variant | MODERATE | >0.05 |
| chr04 | 138,129,978 | C | T | MHC class II antigen, DQ beta 1 chain-like | missense_variant | MODERATE | >0.05 |
| chr04 | 138,527,717 | G | A | C4H6orf10 | missense_variant | MODERATE | >0.05 |
| chr04 | 138,532,683 | T | C | C4H6orf10 | missense_variant | MODERATE | >0.05 |
| chr04 | 138,547,041 | G | C | C4H6orf10 | missense_variant | MODERATE | >0.05 |
| chr04 | 138,728,040 | C | T | EGFL8 | missense_variant | MODERATE | >0.05 |
| chr04 | 138,764,052 | A | G | LOC101864936 | missense_variant | MODERATE | >0.05 |
| chr04 | 138,777,330 | G | A | ATF6B | missense_variant | MODERATE | >0.05 |
| chr04 | 138,832,450 | C | T | TNXB | missense_variant | MODERATE | >0.05 |
| chr04 | 138,832,455 | G | A | TNXB | missense_variant | MODERATE | >0.05 |
| chr04 | 138,832,656 | T | G | TNXB | missense_variant | MODERATE | >0.05 |
| chr04 | 138,838,226 | A | T | TNXB | missense_variant | MODERATE | >0.05 |
| chr04 | 138,842,061 | G | A | TNXB | missense_variant | MODERATE | >0.05 |
| chr04 | 138,854,321 | C | A | steroid 21-hydroxylase | missense_variant | MODERATE | >0.05 |
| chr05 | 56,234,348 | C | A | FRAS1 | missense_variant | MODERATE | >0.05 |
| chr05 | 60,588,329 | G | A | PF4 | missense_variant | MODERATE | >0.05 |
| chr05 | 63,898,042 | C | T | UTP3 | missense_variant | MODERATE | >0.05 |
| chr05 | 64,269,281 | G | A | not identified | missense_variant | MODERATE | >0.05 |
| chr05 | 64,269,359 | C | G | not identified | missense_variant | MODERATE | >0.05 |
| chr05 | 65,731,138 | A | G | UGT2B20 | missense_variant | MODERATE | >0.05 |
| chr05 | 163,236,012 | A | G | LOC102130113 protein theta pseudogene | missense_variant | MODERATE | **2.00 x 10^-04^** |
| chr05 | 163,262,889 | G | A | LOC102130113 protein theta pseudogene | missense_variant | MODERATE | **2.00 x 10^-04^** |
| chr05 | 163,263,047 | G | T | LOC102130113 protein theta pseudogene | missense_variant | MODERATE | **2.00 x 10^-04^** |
| chr05 | 163,263,341 | C | G | LOC102130113 protein theta pseudogene | missense_variant | MODERATE | **2.00 x 10^-04^** |
| chr05 | 185,530,706 | G | C | not identified | missense_variant | MODERATE | >0.05 |
| chr05 | 185,531,796 | G | T | not identified | missense_variant | MODERATE | >0.05 |
| chr06 | 75,148,064 | G | A | CRHBP | missense_variant | MODERATE | >0.05 |
| chr06 | 75,231,433 | G | A | AGGF1 | missense_variant | MODERATE | >0.05 |
| chr06 | 75,248,052 | G | A | AGGF1 | missense_variant | MODERATE | >0.05 |
| chr06 | 75,261,782 | C | T | ZBED3 | missense_variant | MODERATE | >0.05 |
| chr06 | 150,178,899 | G | A | IRGM | missense_variant | MODERATE | >0.05 |
| chr06 | 150,180,713 | G | A | IRGM | splice_donor_variant | HIGH | >0.05 |
| chr06 | 150,316,973 | G | A | TNIP1 | missense_variant | MODERATE | >0.05 |
| chr06 | 150,317,045 | C | T | TNIP1 | missense_variant | MODERATE | >0.05 |
| chr06 | 150,583,542 | G | A | SLC36A3 | missense_variant | MODERATE | **4.70 x 10^-02^** |
| chr06 | 160,063,849 | A | G | ATP10B | missense_variant | MODERATE | **4.30 x 10^-02^** |
| chr06 | 168,273,121 | G | A | SLIT3 | missense_variant | MODERATE | >0.05 |
| chr07 | 50,444,410 | C | G | GOLGA6B | missense_variant | MODERATE | >0.05 |
| chr07 | 57,688,916 | G | C | not identified | missense_variant | MODERATE | >0.05 |
| chr07 | 63,486,883 | T | C | golgin subfamily A member 2 | missense_variant | MODERATE | >0.05 |
| chr07 | 65,190,822 | T | C | ZSCAN2 | missense_variant | MODERATE | >0.05 |
| chr07 | 65,753,686 | G | A | AKAP13 | missense_variant | MODERATE | >0.05 |
| chr07 | 65,759,160 | G | C | AKAP13 | missense_variant | MODERATE | >0.05 |
| chr07 | 65,836,325 | G | A | AKAP13 | missense_variant | MODERATE | >0.05 |
| chr07 | 65,855,383 | G | T | AKAP13 | missense_variant | MODERATE | >0.05 |
| chr07 | 65,898,603 | C | G | AKAP13 | missense_variant | MODERATE | >0.05 |
| chr07 | 65,907,277 | A | T | AKAP13 | missense_variant | MODERATE | >0.05 |
| chr07 | 65,907,333 | G | C | AKAP13 | missense_variant | MODERATE | >0.05 |
| chr07 | 65,909,871 | A | G | AKAP13 | missense_variant | MODERATE | >0.05 |
| chr07 | 65,918,208 | G | A | AKAP13 | missense_variant | MODERATE | >0.05 |
| chr07 | 109,279,238 | C | T | FAM179B | missense_variant | MODERATE | >0.05 |
| chr07 | 126,110,246 | A | G | SNAPC1 | missense_variant | MODERATE | >0.05 |
| chr07 | 130,947,451 | G | A | not identified | missense_variant | MODERATE | >0.05 |
| chr07 | 153,396,359 | G | A | PTPN21 | missense_variant | MODERATE | >0.05 |
| chr07 | 154,818,108 | G | T | EFCAB11 | missense_variant | MODERATE | >0.05 |
| chr07 | 158,322,435 | C | G | BTBD7 | missense_variant | MODERATE | >0.05 |
| chr07 | 160,526,770 | T | C | SYNE3 | missense_variant | MODERATE | >0.05 |
| chr07 | 160,527,046 | G | T | SYNE3 | missense_variant | MODERATE | >0.05 |
| chr07 | 160,530,307 | G | C | SYNE3 | missense_variant | MODERATE | >0.05 |
| chr07 | 160,531,723 | C | T | SYNE3 | missense_variant | MODERATE | >0.05 |
| chr07 | 170,320,745 | G | C | AHNAK2 | missense_variant | MODERATE | >0.05 |
| chr07 | 170,323,815 | G | A | AHNAK2 | missense_variant | MODERATE | >0.05 |
| chr07 | 170,514,683 | G | A | JAG2 | missense_variant | MODERATE | >0.05 |
| chr07 | 170,540,203 | C | G | NUDT14 | missense_variant | MODERATE | >0.05 |
| chr07 | 171,227,695 | G | A | not identified | missense_variant | MODERATE | >0.05 |
| chr07 | 171,544,777 | A | G | not identified | missense_variant | MODERATE | >0.05 |
| chr08 | 27,967,183 | A | G | EPHX2 | missense_variant | MODERATE | >0.05 |
| chr08 | 110,560,986 | G | A | PKHD1L1 | missense_variant | MODERATE | >0.05 |
| chr08 | 110,568,447 | G | A | PKHD1L1 | missense_variant | MODERATE | >0.05 |
| chr09 | 21,827,574 | A | C | calcyclin-binding protein-like | missense_variant | MODERATE | >0.05 |
| chr09 | 22,118,265 | A | G | protein AF-10 | missense_variant | MODERATE | >0.05 |
| chr09 | 41,371,228 | G | C | BMS1 | missense_variant | MODERATE | >0.05 |
| chr09 | 41,418,933 | G | A | BMS1 | missense_variant | MODERATE | >0.05 |
| chr09 | 41,424,478 | G | C | BMS1 | missense_variant | MODERATE | >0.05 |
| chr09 | 46,030,889 | C | T | FAM170B | missense_variant | MODERATE | >0.05 |
| chr09 | 46,233,682 | G | C | WDFY4 | missense_variant | MODERATE | >0.05 |
| chr09 | 46,270,002 | C | A | WDFY4 | missense_variant | MODERATE | >0.05 |
| chr09 | 46,303,295 | C | T | WDFY4 | missense_variant | MODERATE | >0.05 |
| chr09 | 46,303,514 | C | T | WDFY4 | missense_variant | MODERATE | >0.05 |
| chr09 | 46,804,824 | C | G | FRMPD2 | missense_variant | MODERATE | >0.05 |
| chr09 | 46,814,211 | A | T | FRMPD2 | missense_variant | MODERATE | >0.05 |
| chr09 | 47,072,757 | G | A | id373136 | splice_donor_variant | HIGH | >0.05 |
| chr09 | 72,417,663 | C | T | REEP3 | missense_variant | MODERATE | >0.05 |
| chr09 | 72,852,238 | G | A | JMJD1C | missense_variant | MODERATE | >0.05 |
| chr09 | 73,364,505 | T | G | talanin-like | missense_variant | MODERATE | >0.05 |
| chr09 | 73,364,517 | A | C | talanin-like | missense_variant | MODERATE | >0.05 |
| chr09 | 74,112,846 | C | T | ARID5B | missense_variant | MODERATE | >0.05 |
| chr09 | 86,068,691 | G | A | CGRRF1 | missense_variant | MODERATE | >0.05 |
| chr09 | 87,785,306 | C | T | LIPA | missense_variant | MODERATE | >0.05 |
| chr09 | 87,962,237 | T | C | IFIT5 | missense_variant | MODERATE | >0.05 |
| chr09 | 88,241,215 | A | G | PANK1 | missense_variant | MODERATE | >0.05 |
| chr09 | 88,265,477 | C | G | KIF20B | missense_variant | MODERATE | >0.05 |
| chr09 | 94,856,012 | C | A | ZNF518A | missense_variant | MODERATE | >0.05 |
| chr09 | 94,857,464 | A | G | ZNF518A | missense_variant | MODERATE | >0.05 |
| chr09 | 94,858,683 | A | C | ZNF518A | missense_variant | MODERATE | >0.05 |
| chr09 | 94,904,345 | C | T | BLNK | missense_variant | MODERATE | >0.05 |
| chr09 | 96,216,264 | A | G | RRP12 | missense_variant | MODERATE | >0.05 |
| chr09 | 96,384,987 | A | G | ANKRD2 | missense_variant | MODERATE | >0.05 |
| chr09 | 97,044,299 | A | G | R3HCC1L | missense_variant | MODERATE | >0.05 |
| chr09 | 97,044,829 | T | G | R3HCC1L | missense_variant | MODERATE | >0.05 |
| chr09 | 97,045,774 | G | A | R3HCC1L | missense_variant | MODERATE | >0.05 |
| chr09 | 97,079,529 | G | A | LOXL4 | missense_variant | MODERATE | >0.05 |
| chr09 | 97,219,132 | C | T | PYROXD2 | missense_variant | MODERATE | >0.05 |
| chr09 | 97,253,175 | T | C | HPS1 | missense_variant | MODERATE | >0.05 |
| chr09 | 98,553,212 | G | A | ENTPD7 | missense_variant | MODERATE | >0.05 |
| chr09 | 98,614,149 | C | G | cytochrome c oxidase assembly protein COX15 homolog | missense_variant | MODERATE | >0.05 |
| chr09 | 98,614,195 | C | T | cytochrome c oxidase assembly protein COX15 homolog | missense_variant | MODERATE | >0.05 |
| chr09 | 98,614,216 | T | C | cytochrome c oxidase assembly protein COX15 homolog | missense_variant | MODERATE | >0.05 |
| chr09 | 98,614,327 | C | G | cytochrome c oxidase assembly protein COX15 homolog | missense_variant | MODERATE | >0.05 |
| chr09 | 99,427,315 | C | T | SEC31B | missense_variant | MODERATE | >0.05 |
| chr09 | 99,432,957 | C | T | SEC31B | missense_variant | MODERATE | >0.05 |
| chr09 | 99,930,955 | G | A | SEMA4G | missense_variant | MODERATE | >0.05 |
| chr09 | 99,935,963 | T | C | C9H10orf2 | missense_variant | MODERATE | >0.05 |
| chr09 | 99,936,253 | C | G | C9H10orf2 | missense_variant | MODERATE | >0.05 |
| chr09 | 99,936,404 | C | A | C9H10orf2 | missense_variant | MODERATE | >0.05 |
| chr09 | 99,938,431 | G | A | C9H10orf2 | missense_variant | MODERATE | >0.05 |
| chr09 | 99,953,791 | G | A | LZTS2 | missense_variant | MODERATE | >0.05 |
| chr09 | 99,957,121 | G | A | LZTS2 | missense_variant | MODERATE | >0.05 |
| chr09 | 100,013,339 | G | A | KAZALD1 | missense_variant | MODERATE | >0.05 |
| chr09 | 100,528,141 | G | A | POLL | missense_variant | MODERATE | >0.05 |
| chr09 | 109,036,852 | CG | TA | XPNPEP1 | missense_variant | MODERATE | >0.05 |
| chr09 | 111,479,327 | C | T | guanylate cyclase 2G-like | missense_variant | MODERATE | >0.05 |
| chr09 | 111,482,483 | C | T | guanylate cyclase 2G-like | missense_variant | MODERATE | >0.05 |
| chr09 | 111,483,543 | G | A | guanylate cyclase 2G-like | missense_variant | MODERATE | >0.05 |
| chr09 | 111,486,546 | C | T | guanylate cyclase 2G-like | missense_variant | MODERATE | >0.05 |
| chr09 | 111,504,161 | C | T | guanylate cyclase 2G-like | missense_variant | MODERATE | >0.05 |
| chr09 | 111,504,204 | T | C | guanylate cyclase 2G-like | missense_variant | MODERATE | >0.05 |
| chr10 | 2,459,278 | C | T | not identified | missense_variant | MODERATE | >0.05 |
| chr10 | 2,459,289 | G | A | not identified | missense_variant | MODERATE | >0.05 |
| chr10 | 5,819,110 | G | A | NUP50 | missense_variant | MODERATE | **1.60 x 10^-02^** |
| chr10 | 6,077,347 | C | T | PHF21B | missense_variant | MODERATE | **1.10 x 10^-02^** |
| chr10 | 6,169,071 | G | A | ARHGAP8 | missense_variant | MODERATE | **2.30 x 10^-02^** |
| chr10 | 32,916,540 | C | G | MICAL3 | missense_variant | MODERATE | >0.05 |
| chr10 | 32,962,898 | C | G | BID | missense_variant | MODERATE | >0.05 |
| chr10 | 33,716,824 | C | T | IL17RA | missense_variant | MODERATE | >0.05 |
| chr10 | 33,717,116 | C | T | IL17RA | missense_variant | MODERATE | >0.05 |
| chr10 | 33,995,323 | G | A | GAB4 | missense_variant | MODERATE | >0.05 |
| chr10 | 33,995,617 | T | C | GAB4 | missense_variant | MODERATE | >0.05 |
| chr10 | 38,896,301 | T | C | GZF1 | missense_variant | MODERATE | **2.40 x 10^-02^** |
| chr10 | 48,561,206 | G | C | SEL1L2 | splice_acceptor_variant | HIGH | **2.20 x 10^-02^** |
| chr10 | 48,585,936 | C | T | SEL1L2 | missense_variant | MODERATE | >0.05 |
| chr10 | 48,601,380 | G | A | SEL1L2 | missense_variant | MODERATE | >0.05 |
| chr10 | 48,601,426 | C | T | SEL1L2 | missense_variant | MODERATE | >0.05 |
| chr10 | 48,602,047 | A | G | SEL1L2 | missense_variant | MODERATE | **2.20 x 10^-02^** |
| chr10 | 48,604,843 | A | G | SEL1L2 | missense_variant | MODERATE | **4.30 x 10^-02^** |
| chr10 | 48,606,368 | C | G | SEL1L2 | missense_variant | MODERATE | **2.20 x 10^-02^** |
| chr10 | 48,606,397 | G | A | SEL1L2 | missense_variant | MODERATE | **2.20 x 10^-02^** |
| chr10 | 57,187,022 | C | T | PROKR2 | missense_variant | MODERATE | >0.05 |
| chr10 | 59,310,794 | G | A | DDRGK1 | missense_variant | MODERATE | >0.05 |
| chr10 | 59,369,353 | A | G | FASTKD5 | missense_variant | MODERATE | >0.05 |
| chr10 | 59,948,910 | C | T | TMC2 | missense_variant | MODERATE | >0.05 |
| chr10 | 64,714,948 | T | C | CDK5RAP1 | missense_variant | MODERATE | >0.05 |
| chr10 | 64,719,575 | C | G | CDK5RAP1 | missense_variant | MODERATE | >0.05 |
| chr10 | 72,837,959 | C | T | ZHX3 | missense_variant | MODERATE | **1.40 x 10^-02^** |
| chr10 | 72,839,745 | C | T | ZHX3 | missense_variant | MODERATE | **3.10 x 10^-02^** |
| chr10 | 73,000,757 | C | T | EMILIN3 | missense_variant | MODERATE | >0.05 |
| chr10 | 76,039,089 | C | T | R3HDML | missense_variant | MODERATE | >0.05 |
| chr10 | 76,481,974 | C | T | KCNK15 | missense_variant | MODERATE | >0.05 |
| chr10 | 88,277,201 | G | A | CASS4 | missense_variant | MODERATE | **2.60 x 10^-02^** |
| chr10 | 88,291,159 | G | A | CASS4 | missense_variant | MODERATE | >0.05 |
| chr10 | 88,291,557 | A | T | CASS4 | missense_variant | MODERATE | >0.05 |
| chr10 | 88,345,554 | C | T | GCNT7 | missense_variant | MODERATE | >0.05 |
| chr10 | 94,285,768 | T | C | LAMA5 | missense_variant | MODERATE | >0.05 |
| chr10 | 96,449,358 | A | G | MYT1 | stop_lost | HIGH | >0.05 |
| chr11 | 4,764,284 | C | T | DYRK4 | missense_variant | MODERATE | >0.05 |
| chr11 | 8,774,958 | C | G | CLEC4E | missense_variant | MODERATE | >0.05 |
| chr11 | 9,142,274 | A | G | A2ML1 | missense_variant | MODERATE | >0.05 |
| chr11 | 11,704,868 | C | T | taste receptor type 2 member 31 | missense_variant | MODERATE | >0.05 |
| chr11 | 13,414,234 | G | A | DDX47 | missense_variant | MODERATE | >0.05 |
| chr11 | 28,407,439 | G | A | MRPS35 | missense_variant | MODERATE | >0.05 |
| chr11 | 28,450,808 | A | G | MANSC4 | missense_variant | MODERATE | >0.05 |
| chr11 | 97,985,059 | C | T | PLXNC1 | missense_variant | MODERATE | >0.05 |
| chr11 | 102,322,746 | C | T | IKBIP | missense_variant | MODERATE | >0.05 |
| chr11 | 112,931,950 | G | T | LOC102118081 polyadenylate-binding protein 1 pseudogene | missense_variant | MODERATE | >0.05 |
| chr11 | 112,932,587 | G | T | LOC102118081 polyadenylate-binding protein 1 pseudogene | missense_variant | MODERATE | >0.05 |
| chr11 | 112,932,818 | A | C | LOC102118081 polyadenylate-binding protein 1 pseudogene | missense_variant | MODERATE | >0.05 |
| chr11 | 112,932,833 | C | G | LOC102118081 polyadenylate-binding protein 1 pseudogene | missense_variant | MODERATE | >0.05 |
| chr11 | 112,933,267 | T | C | LOC102118081 polyadenylate-binding protein 1 pseudogene | missense_variant | MODERATE | >0.05 |
| chr12 | 94,675,338 | G | C | PARD3B | missense_variant | MODERATE | >0.05 |
| chr12 | 119,980,161 | A | T | SP110 nuclear body protein | missense_variant | MODERATE | >0.05 |
| chr12 | 119,980,468 | A | G | SP110 nuclear body protein | missense_variant | MODERATE | >0.05 |
| chr12 | 120,059,608 | A | G | SP140 | missense_variant | MODERATE | >0.05 |
| chr12 | 120,063,781 | A | T | SP140 | missense_variant | MODERATE | >0.05 |
| chr12 | 120,178,776 | G | A | SP140L | missense_variant | MODERATE | >0.05 |
| chr12 | 123,534,466 | T | G | UGT1A5 | missense_variant | MODERATE | >0.05 |
| chr12 | 127,924,249 | G | A | SCLY | missense_variant | MODERATE | >0.05 |
| chr12 | 128,009,447 | G | A | FAM132B | missense_variant | MODERATE | >0.05 |
| chr13 | 10,618,814 | G | A | C13H2orf40 | missense_variant | MODERATE | >0.05 |
| chr13 | 16,364,499 | A | G | FBLN7 | missense_variant | MODERATE | >0.05 |
| chr13 | 17,127,304 | G | A | IL-36G | missense_variant | MODERATE | >0.05 |
| chr13 | 17,132,994 | A | T | IL-36G | missense_variant | MODERATE | >0.05 |
| chr13 | 17,133,365 | A | G | IL-36G 3'UTR | 3'UTR_variant | unknown | **4.40 x 10^-03^** |
| chr13 | 17,133,758 | A | G | IL-36G 3'UTR | 3'UTR_variant | unknown | **3.20 x 10^-03^** |
| chr13 | 24,424,460 | G | A | KCMF1 | missense_variant | MODERATE | >0.05 |
| chr13 | 35,353,124 | T | C | DOK1 | missense_variant | MODERATE | >0.05 |
| chr13 | 35,448,396 | C | A | MOGS | missense_variant | MODERATE | **3.10 x 10^-02^** |
| chr13 | 35,485,473 | T | C | RTKN | missense_variant | MODERATE | >0.05 |
| chr13 | 35,489,941 | G | A | WDR54 | missense_variant | MODERATE | >0.05 |
| chr13 | 35,497,885 | C | A | C13H2orf81 | missense_variant | MODERATE | >0.05 |
| chr13 | 35,499,531 | G | A | not identified | stop_gained | HIGH | **3.10 x 10^-02^** |
| chr13 | 36,192,509 | T | C | TPRKB | missense_variant | MODERATE | >0.05 |
| chr13 | 36,389,913 | T | C | ALMS1 | missense_variant | MODERATE | >0.05 |
| chr13 | 36,393,072 | G | T | ALMS1 | missense_variant | MODERATE | >0.05 |
| chr13 | 54,733,416 | A | G | CCDC88A | missense_variant | MODERATE | >0.05 |
| chr13 | 55,032,929 | AG | GA | RTN4 | missense_variant | MODERATE | >0.05 |
| chr13 | 56,563,656 | A | G | ferritin heavy chain pseudogene | missense_variant | MODERATE | >0.05 |
| chr14 | 848,323 | C | A | MUC6 | missense_variant | MODERATE | >0.05 |
| chr14 | 3,458,002 | G | A | actin-85C-like | missense_variant | MODERATE | >0.05 |
| chr14 | 3,714,675 | G | A | SHANK2 | missense_variant | MODERATE | >0.05 |
| chr14 | 3,735,182 | C | T | SHANK2 | missense_variant | MODERATE | >0.05 |
| chr14 | 3,747,319 | G | A | SHANK2 | missense_variant | MODERATE | >0.05 |
| chr14 | 4,376,550 | A | C | not identified | missense_variant | MODERATE | >0.05 |
| chr14 | 4,396,359 | G | T | PPFIA1 | missense_variant | MODERATE | >0.05 |
| chr14 | 12,992,696 | A | G | VWCE | missense_variant | MODERATE | >0.05 |
| chr14 | 13,053,314 | C | G | pepsin A | missense_variant | MODERATE | >0.05 |
| chr14 | 13,354,161 | C | T | TMEM132A | missense_variant | MODERATE | >0.05 |
| chr14 | 13,478,017 | T | C | MS4A10 | stop_lost | HIGH | >0.05 |
| chr14 | 13,481,656 | G | C | MS4A10 | missense_variant | MODERATE | >0.05 |
| chr14 | 13,537,001 | GT | CC | MS4A18 | missense_variant | MODERATE | >0.05 |
| chr14 | 13,742,769 | G | A | MS4A12 | missense_variant | MODERATE | >0.05 |
| chr14 | 13,743,064 | G | A | MS4A12 | missense_variant | MODERATE | >0.05 |
| chr14 | 13,743,113 | G | T | MS4A12 | missense_variant | MODERATE | >0.05 |
| chr14 | 13,747,732 | TG | CA,CG | MS4A12 | missense_variant | MODERATE | >0.05 |
| chr14 | 13,748,540 | T | C | MS4A12 | missense_variant | MODERATE | >0.05 |
| chr14 | 13,748,564 | G | A | MS4A12 | missense_variant | MODERATE | >0.05 |
| chr14 | 13,751,932 | T | A | MS4A12 | missense_variant | MODERATE | >0.05 |
| chr14 | 13,891,885 | T | G | membrane-spanning 4-domains subfamily A member 6A-like | missense_variant | MODERATE | >0.05 |
| chr14 | 13,896,808 | G | A | membrane-spanning 4-domains subfamily A member 6A-like | missense_variant | MODERATE | >0.05 |
| chr14 | 31,961,178 | G | A | PAMR1 | missense_variant | MODERATE | >0.05 |
| chr14 | 59,853,356 | G | C | EIF3F | missense_variant | MODERATE | >0.05 |
| chr14 | 59,853,592 | G | A | EIF3F | missense_variant | MODERATE | >0.05 |
| chr14 | 59,853,610 | G | A | EIF3F | missense_variant | MODERATE | >0.05 |
| chr14 | 59,853,644 | G | C | EIF3F | missense_variant | MODERATE | >0.05 |
| chr14 | 59,853,664 | C | G | EIF3F | missense_variant | MODERATE | >0.05 |
| chr14 | 64,967,708 | G | C | olfactory receptor 51I2 | stop_gained | HIGH | >0.05 |
| chr14 | 77,876,668 | A | G | LOC102128847 beta-citrylglutamate synthase B pseudogene | missense_variant | MODERATE | **2.40 x 10^-03^** |
| chr14 | 85,357,655 | G | C | putative tripartite motif-containing protein 64B | missense_variant | MODERATE | >0.05 |
| chr14 | 87,584,477 | T | G | LOC102118617 40S ribosomal protein S13 pseudogene | missense_variant | MODERATE | >0.05 |
| chr14 | 104,762,896 | G | A | DDX10 | missense_variant | MODERATE | **2.90 x 10^-02^** |
| chr14 | 111,944,124 | T | A | not identified | missense_variant | MODERATE | >0.05 |
| chr14 | 121,401,037 | C | A | STT3A | missense_variant | MODERATE | >0.05 |
| chr15 | 10,947,220 | A | G | GOLGA2 | missense_variant | MODERATE | >0.05 |
| chr15 | 11,649,631 | C | G | GPR144 | missense_variant | MODERATE | >0.05 |
| chr15 | 28,763,705 | C | T | FAM206A | missense_variant | MODERATE | >0.05 |
| chr15 | 64,021,055 | A | T | FREM1 | missense_variant | MODERATE | >0.05 |
| chr15 | 96,905,732 | C | T | C15H9orf153 | missense_variant | MODERATE | >0.05 |
| chr15 | 106,325,548 | A | G | SPATA31E1 | missense_variant | MODERATE | >0.05 |
| chr16 | 3,122,135 | G | A | olfactory receptor 1D2 | missense_variant | MODERATE | >0.05 |
| chr16 | 4,575,143 | C | T | GGT6 | missense_variant | MODERATE | >0.05 |
| chr16 | 4,575,320 | A | G | GGT6 | missense_variant | MODERATE | >0.05 |
| chr16 | 4,575,424 | C | T | GGT6 | missense_variant | MODERATE | >0.05 |
| chr16 | 4,575,458 | G | T | GGT6 | missense_variant | MODERATE | >0.05 |
| chr16 | 4,576,114 | C | T | GGT6 | missense_variant | MODERATE | >0.05 |
| chr16 | 10,048,296 | G | A | GLP2R | missense_variant | MODERATE | >0.05 |
| chr16 | 10,498,978 | G | A | MYH13 | missense_variant | MODERATE | **5.40 x 10^-03^** |
| chr16 | 10,573,814 | A | G | MYH8 | splice_donor_variant | HIGH | **5.40 x 10^-03^** |
| chr16 | 15,943,014 | T | C | TBC1D27 | missense_variant | MODERATE | >0.05 |
| chr16 | 16,232,654 | T | G | ZNF624 | missense_variant | MODERATE | >0.05 |
| chr16 | 21,171,686 | A | G | C16H17orf51 | missense_variant | MODERATE | >0.05 |
| chr16 | 21,171,808 | C | A | C16H17orf51 | missense_variant | MODERATE | >0.05 |
| chr16 | 21,171,812 | A | T | C16H17orf51 | missense_variant | MODERATE | >0.05 |
| chr16 | 21,171,820 | T | C | C16H17orf51 | missense_variant | MODERATE | >0.05 |
| chr16 | 21,171,849 | CG | GT | C16H17orf51 | missense_variant | MODERATE | >0.05 |
| chr16 | 21,171,889 | G | A | C16H17orf51 | missense_variant | MODERATE | >0.05 |
| chr16 | 21,186,302 | C | A | C16H17orf51 | missense_variant | MODERATE | >0.05 |
| chr16 | 21,186,367 | C | G | C16H17orf51 | missense_variant | MODERATE | >0.05 |
| chr16 | 21,186,422 | G | A | C16H17orf51 | missense_variant | MODERATE | >0.05 |
| chr16 | 21,186,488 | C | T | C16H17orf51 | missense_variant | MODERATE | **4.30 x 10^-02^** |
| chr16 | 21,186,493 | C | G | C16H17orf51 | missense_variant | MODERATE | >0.05 |
| chr16 | 21,186,542 | C | G | C16H17orf51 | missense_variant | MODERATE | >0.05 |
| chr16 | 21,186,563 | T | C | C16H17orf51 | missense_variant | MODERATE | >0.05 |
| chr16 | 21,186,614 | A | G | C16H17orf51 | missense_variant | MODERATE | >0.05 |
| chr16 | 21,186,641 | TG | CC | C16H17orf51 | missense_variant | MODERATE | >0.05 |
| chr16 | 21,186,673 | G | C | C16H17orf51 | missense_variant | MODERATE | >0.05 |
| chr16 | 27,458,663 | T | C | mucin-5AC-like | missense_variant | MODERATE | >0.05 |
| chr16 | 28,317,971 | C | T | ATAD5 | missense_variant | MODERATE | >0.05 |
| chr16 | 50,627,718 | C | G | PLXDC1 | missense_variant | MODERATE | >0.05 |
| chr16 | 51,593,012 | A | G | GSDMA | missense_variant | MODERATE | >0.05 |
| chr16 | 55,754,527 | T | C | NAGS | missense_variant | MODERATE | >0.05 |
| chr16 | 56,043,360 | C | A | SLC4A1 | missense_variant | MODERATE | >0.05 |
| chr16 | 56,197,228 | C | A | GPATCH8 | missense_variant | MODERATE | >0.05 |
| chr16 | 56,726,173 | C | T | GFAP | missense_variant | MODERATE | >0.05 |
| chr16 | 56,726,394 | G | A | GFAP | missense_variant | MODERATE | >0.05 |
| chr16 | 57,054,252 | G | A | FMNL1 | missense_variant | MODERATE | >0.05 |
| chr16 | 57,099,508 | G | A | MAP3K14 | missense_variant | MODERATE | >0.05 |
| chr16 | 57,557,194 | C | T | LRRC37A3 | missense_variant | MODERATE | >0.05 |
| chr16 | 57,557,244 | C | T | LRRC37A3 | missense_variant | MODERATE | >0.05 |
| chr16 | 57,558,915 | C | G | LRRC37A3 | missense_variant | MODERATE | >0.05 |
| chr16 | 57,558,925 | G | C | LRRC37A3 | missense_variant | MODERATE | >0.05 |
| chr18 | 50,393,251 | G | A | TRAPPC8 | missense_variant | MODERATE | >0.05 |
| chr18 | 50,678,195 | G | T | TTR | missense_variant | MODERATE | >0.05 |
| chr18 | 50,725,397 | G | C | DSG2 | missense_variant | MODERATE | >0.05 |
| chr18 | 51,183,918 | G | A | DSC1 | missense_variant | MODERATE | **1.20 x 10^-02^** |
| chr18 | 51,250,991 | G | A | DSC2 | missense_variant | MODERATE | **2.60 x 10^-02^** |
| chr18 | 58,361,071 | T | C | LAMA3 | missense_variant | MODERATE | >0.05 |
| chr18 | 59,286,196 | T | C | RBBP8 | missense_variant | MODERATE | >0.05 |
| chr18 | 68,195,996 | C | T | LAMA1 | missense_variant | MODERATE | **1.60 x 10^-02^** |
| chr18 | 75,705,129 | C | T | ANKRD30B | missense_variant | MODERATE | >0.05 |
| chr18 | 75,705,147 | A | G | ANKRD30B | missense_variant | MODERATE | >0.05 |
| chr19 | 3,561,656 | G | A | GIPC3 | missense_variant | MODERATE | **4.90 x 10^-02^** |
| chr19 | 3,562,100 | A | G | GIPC3 | missense_variant | MODERATE | >0.05 |
| chr19 | 6,585,441 | A | G | not identified | missense_variant | MODERATE | >0.05 |
| chr19 | 9,152,548 | T | C | MUC16 | missense_variant | MODERATE | >0.05 |
| chr19 | 9,270,696 | T | C | MUC16 | missense_variant | MODERATE | >0.05 |
| chr19 | 13,171,620 | G | C | CALR | missense_variant | MODERATE | >0.05 |
| chr19 | 16,789,882 | G | A | C19H19orf44 | missense_variant | MODERATE | >0.05 |
| chr19 | 17,874,781 | G | A | FAM129C | missense_variant | MODERATE | >0.05 |
| chr19 | 17,874,872 | C | G | FAM129C | missense_variant | MODERATE | >0.05 |
| chr19 | 17,880,052 | G | T | FAM129C | stop_gained | HIGH | >0.05 |
| chr19 | 17,883,351 | G | A | FAM129C | missense_variant | MODERATE | >0.05 |
| chr19 | 18,076,978 | G | A | MAP1S | missense_variant | MODERATE | >0.05 |
| chr19 | 36,277,946 | T | A | NPHS1 | missense_variant | MODERATE | >0.05 |
| chr19 | 36,278,561 | T | G | NPHS1 | missense_variant | MODERATE | >0.05 |
| chr19 | 36,290,775 | C | T | NPHS1 | missense_variant | MODERATE | >0.05 |
| chr19 | 43,625,407 | C | T | not identified | missense_variant | MODERATE | >0.05 |
| chr19 | 49,975,568 | C | T | HRC | missense_variant | MODERATE | >0.05 |
| chr19 | 49,976,596 | G | A | HRC | missense_variant | MODERATE | >0.05 |
| chr19 | 49,976,758 | C | T | HRC | missense_variant | MODERATE | >0.05 |
| chr19 | 49,976,951 | G | A | HRC | missense_variant | MODERATE | >0.05 |
| chr19 | 50,813,096 | C | T | ATF5 | missense_variant | MODERATE | >0.05 |
| chr20 | 15,122,792 | C | T | PLA2G10 | missense_variant | MODERATE | >0.05 |
| chr20 | 15,158,200 | C | T | nuclear pore complex-interacting protein family member B11-like | missense_variant | MODERATE | >0.05 |
| chr20 | 15,169,027 | T | C | nuclear pore complex-interacting protein family member B11-like | missense_variant | MODERATE | >0.05 |
| chr20 | 15,619,408 | C | T | C20H16orf45 | missense_variant | MODERATE | >0.05 |
| chr20 | 34,158,109 | T | G | ankyrin repeat domain-containing protein 26-like | missense_variant | MODERATE | >0.05 |
| chr20 | 34,194,478 | A | G | ankyrin repeat domain-containing protein 26-like | missense_variant | MODERATE | >0.05 |
| chr20 | 34,201,240 | T | A | ankyrin repeat domain-containing protein 26-like | missense_variant | MODERATE | >0.05 |
| chr20 | 34,386,782 | C | T | MYLK3 | missense_variant | MODERATE | >0.05 |
| chr20 | 34,402,743 | C | G | MYLK3 | missense_variant | MODERATE | >0.05 |
| chr20 | 46,322,896 | C | G | PRSS54 | missense_variant | MODERATE | >0.05 |
| chr20 | 58,054,381 | T | C | NOB1 | missense_variant | MODERATE | >0.05 |
| chr20 | 62,166,233 | G | A | FUK | missense_variant | MODERATE | >0.05 |
| chrX | 35,742,548 | T | C | FAM47D | missense_variant | MODERATE | >0.05 |
| chrX | 36,010,761 | A | C | LOC102122771 peptidyl-prolyl cis-trans isomerase D pseudogene | missense_variant | MODERATE | >0.05 |
| chrX | 50,696,215 | A | G | MAGED4 (melanoma-associated antigen D4) | missense_variant | MODERATE | **7.90 x 10^-03^** |
| chrX | 78,112,429 | A | G | HMGN5 | missense_variant | MODERATE | **4.80 x 10^-02^** |
| chrX | 98,643,427 | G | A | ARMCX4 | missense_variant | MODERATE | >0.05 |

**Legend of supplementary table S2a.**

^1^ locations on chromosomes (*Macaca fascicularis* RefSeq assembly GCF_000364345.1)

^2^ REF: base present in the *Macaca fascicularis* genome (GCF_000364345.1)

^3^ ALT: alternative base at the position

^4^ the effect on the encoded proteins were obtained by using SnpEff4.0e with the *Macaca fascicularis* built-in database ^1^.

^5^ p-value (Fisher's Exact Test), p values <0.05 are in bold and underlined characters.

**Supplementary table S2b: Chromosomal locations of the 424 SNPs studied in 42 animals : number of SNPs per chromosome**.

| #CHROM | SNP  numbers | Gene  numbers | pseudogene numbers  (SNPs number) | pseudogene codes | SNP in  3'UTR | olfactory receptors (SNPs numbers) | Not identified gene numbers (#SNPs numbers) |
| --- | --- | --- | --- | --- | --- | --- | --- |
| chr01 | 34 | 27 | 1 (1) | LOC102131552 |  | 4 (6) | 1(1) |
| chr02 | 12 | 7 |  |  |  |  |  |
| chr03 | 9 | 7 |  |  |  |  |  |
| chr04 | 46 | 28 |  |  |  |  |  |
| chr05 | 12 | 7 | 1(4) | LOC102130113 |  |  | 2(4) |
| chr06 | 11 | 8 |  |  |  |  |  |
| chr07 | 29 | 17 |  |  |  |  | 4(4) |
| chr08 | 3 | 2 |  |  |  |  |  |
| chr09 | 58 | 34 |  |  |  |  |  |
| chr10 | 37 | 23 |  |  |  |  | 1(2) |
| chr11 | 14 | 10 | 1(5) | LOC102118081 |  |  |  |
| chr12 | 9 | 7 |  |  |  |  |  |
| chr13 | 19 | 16 | 1 (1) | LOC102124852 | 2 (IL-36G) |  | 1(1) |
| chr14 | 35 | 21 | 2 (2) | LOC102128847  LOC102118617 |  | 1 (1) | 2(2) |
| chr15 | 6 | 6 |  |  |  |  |  |
| chr16 | 42 | 19 |  |  |  | 1 (1) |  |
| chr18 | 10 | 9 |  |  |  |  |  |
| chr19 | 21 | 11 |  |  |  |  | 2(2) |
| chr20 | 12 | 8 |  |  |  |  |  |
| chrX | 5 | 5 | 1 (1) | LOC102122771 |  |  |  |
| **total=** | 424 | 272 | 7 (14) |  | 2 | 6(8) | 13 (16) |

Remark: there is 394 SNPs in 252 genes encoding clearly identified proteins

**Supplementary table S2c:** List of genes involved in HIV-1 infection and /or replication

| **Symbol of gene** | **HIV related (1)** | **Interaction**  **with HIV proteins (2)** | **HIV replication enhanced by knock-down of the gene (2)** | **References** |
| --- | --- | --- | --- | --- |
| **C4HC6orf1** |  |  | **yes** | ^2^ |
| **DDX10** |  |  | **yes** | ^3^ |
| **MICAL3** |  |  | **yes** | ^4^ |
| **ZNF518A** |  |  | **yes** | ^5^ |
| **UMODL1** |  |  | **yes** | ^4^ |
| **TMEM132A** |  |  | **yes** | ^4^ |
| **PSMA1** |  |  | **yes** | ^4^ |
| **STT3A** |  |  | **yes** | ^2^ |
| **SLC4A1** |  |  | **yes** | ^5^ |
| **IL-36G** |  |  | **yes** | ^6^ |
| **R3HDML** |  |  | **yes** | ^6^ |
| **FCGR2B** |  | downregulates Nef |  | ^7^ |
| **LAMA1** |  | glycoprotein gp120 (inhibits) |  | ^8^ |
| **LAMA3** |  | glycoprotein gp120 (inhibits) |  | ^8^ |
| **LAMA5** |  | glycoprotein gp120 (inhibits) |  | ^8^ |
| **BID** |  | Tat, glycoprotein gp120, retropepsin, Vpr |  | ^9^ |
| **PLA2G10** |  | degradation of the viral membrane (gp120) |  | ^10^ |
| **PF4** |  | glycoprotein gp120 (binds) |  | ^11^ |
| **CALR** |  | glycoprotein gp120, gp160, precursor Rev-Vpr |  | ^12^ |
| **FETUB** |  | glycoprotein gp160 (binds) |  | ^13^ |
| **IRGM** |  | IRGM is a common target of RNA viruses |  | ^14,15^ |
| **MOGS** |  | Nef AND Gag_pol |  | ^16^ |
| **TNIP1** |  | Binds to HIV-1 matrix |  | ^17^ |
| **RPL5** |  | Pr55(Gag), Nef, Gag-Pol,retropepsin,Rev |  | ^16^ |
| **DOK1** |  | retropepsin |  | ^12^ |
| **MYH8** |  | retropepsin (cleaves) |  | ^18^ |
| **MYH13** |  | retropepsin (cleaves) |  | ^18^ |
| **SPTA1** |  | retropepsin (cleaves) |  | ^19^ |
| **EIF3F** |  | Inhibition of HIV-1 replication |  | ^20^ |
| **DDX47** |  | Intercats with Rev |  | ^21^ |
| **TCEA3** |  | Tat |  | ^22^ |
| **HSPG2** |  | Tat |  | ^23^ |
| **TTR** |  | Tat (downregulates) |  | ^24^ |
| **DSC1** |  | Tat downregulates expression of DSC1 |  | ^25^ |
| **TARBP1** |  | TAR (HIV-1) RNA binding protein 1 Tat (regulates) |  | ^26^ |
| **SP140** |  | Interaction with Vif |  | ^27^ |
| **LIPA** | gene expression decreased during the second phase of HIV-1 infection |  |  | ^28^ |
| **HPS1** | gene expression decreased during the second phase of HIV-1 infection |  |  | ^28^ |
| **FRAS1** | polymorphism of FREM1 is associated with resistance against HIV infection |  |  | ^29^ |
| **SLIT3** | Slit2 inhibbits HIV-1 replication in T cells |  |  | ^30^ |
| **SRPK1** | Serine/threonine-protein kinase used by RNA-splicing machinery during viral replication |  |  | ^31^ |

**Supplementary table S2d:** List of genes involved in immune responses, or in HIV-1 infection

| **Symbol of gene** | **Function in the immune system** | **References** |
| --- | --- | --- |
| **IRGM** | autophagy | ^12^ |
| **FCGR2B** | Fc gamma receptor | ^7^ |
| **NCR2** | CD336 natural cytotoxicity triggering receptor | ^32^ |
| **ATF6B** | hepatitis C fight | ^33^ |
| **BLNK** | immune response | ^34^ |
| **PYHIN1** | inflammation | ^35^ |
| **ITGA9**  (Integrin alpha-9) | Immune response | ^36^ |
| **ETV7** | monocyte type 1 IFN response genes | ^37^ |
| **UHRF1BP1** | regulation of nuclear factor kappa-B activation | ^38^ |
| **MS4A10** | related to CD20 | ^39^ |
| **SH2D2A** | T cell-specific adapter protein | ^40^ |
| **MHC class II, DP beta 1 chain** | Presentation of peptide | See commentary (1) |
| **MHC class II, DP alpha 1 chain** | Presentation of peptide | See commentary (1) |
| **MHC class II, DQ beta 1 chain-like** | Presentation of peptide | See commentary (1) |
| **MHC class II, DM beta chain** | Presentation of peptide | See commentary (1) |
| **MHC class II, DO beta chain** | Presentation of peptide | See commentary (1) |
| **MHC class II, DO beta chain** | Presentation of peptide | See commentary (1) |
| **TAP2** | Transport of peptides presented by MCH class I proteins | See commentary (2) |

Commentary (1): although MHC class II region genes were not found to be associated with the control of HIV infection in humans, the MHC class II proteins are required to present antigenic peptides to CD4+ lymphocytes and therefore play a crucial role in the adaptive immune responses against infectious pathogens including HIV (for general review see ^41^). Recently it was reported that HIV-Infected dendritic cells present endogenous MHC Class II-restricted antigens to HIV-Specific CD4+ T Cells ^42^. In SIV-infected rhesus macaques, Sauerman and col. reported that homozygosity for a Conserved MHC Class II DQ-DRB haplotype was associated with rapid disease progression ^43^. Giraldo-Vela and col. reported that two Mamu-DRB1 alleles were enriched in a cohort of SIV-infected rhesus monkey elite controllers ^44^.

Commentary (2) TAP2 (Transporter associated with Antigen Processing) form a complex with TAP1 which is essential for the transportation of peptides from the cytosol to the endoplasmic reticulum where the peptides are loaded in the peptide‐binding grooves of MHC class I proteins (for review see ^45,46^). Antigen-specific CD8 T-cells recognize peptides that are associated with MHC class I proteins. The role of MCH class I gene polymorphism in the control of HIV infection in human was clearly established ^47,48^ (for review ^49^). Mann et al. identified particular MHC class I–TAP genotype combinations associated with a prolonged AIDS-free time, whereas several other class I–TAP combinations were associated with rapid disease progression ^50^. Liu and col. identified a combined influence of polymorphisms in human leukocyte antigen class I and transporter on resistance to HIV-1 infection ^51^. In case of macaque, the control of the SIV infection is associated with the MHC class I genotype in the cynomolgus and rhesus macaque models ^52-60^. To our knowledge, no studies about combined effect of MCH class I and TAP genotypes on SIV infection have been published until now.

**Supplementary table S3:** Detection of 31 variants that show weak probabilities of association with LogPVL (0.01<P<0.05) in 42 SIV-infected cynomolgus macaques.

| **Gene** | **Chr.** | **Location*** | **P-value by exact test** | **Odds ratio** | **P-value** | **95% Cl** |
| --- | --- | --- | --- | --- | --- | --- |
| PHF21B | 10 | 6,077,347 | 1.1 x 10^-2^ | 9,0 | 1.8 x 10^-2^ | 1.359 - Inf |
| DSC1 | 18 | 51,183,918 | 1.2 x 10^-2^ | 4,8 | 2.9 x 10^-2^ | 1.027 - 25.402 |
| ZHX3 | 10 | 72,837,959 | 1.4 x 10^-2^ | 4,3 | 3.3 x 10^-2^ | 0.982 - 20.475 |
| LAMA1 | 18 | 68,195,996 | 1.6 x 10^-2^ | 4,2 | NS | 0.780 - 28.651 |
| LOC101925861 | 10 | 5,819,110 | 1.6 x 10^-2^ | 6,2 | 3.5 x 10^-2^ | 0.997 - 65.910 |
| SEL1L2 | 10 | 48,561,206 | 2.2 x 10^-2^ | 4,7 | 4.7 x 10^-2^ | 0.910 - 31.239 |
|  | 10 | 48,602,047 | 2.2 x 10^-2^ | 4,7 | 4.7 x 10^-2^ | 0.910 - 31.239 |
|  | 10 | 48,606,368 | 2.2 x 10^-2^ | 4,7 | 4.7 x 10^-2^ | 0.910 - 31.239 |
|  | 10 | 48,606,397 | 2.2 x 10^-2^ | 4,7 | 4.7 x 10^-2^ | 0.910 - 31.239 |
| ARHGAP8 | 10 | 6,169,071 | 2.3 x 10^-2^ | 10,9 | 3.7 x 10^-2^ | 1.023 - 523.217 |
| GZF1 | 10 | 38,896,301 | 2.4 x 10^-2^ | 5,9 | 4.9 x 10^-2^ | 0.916 - Inf |
| CASS4 | 10 | 88,277,201 | 2.6 x 10^-2^ | 5,5 | 2.3 x 10^-2^ | 1.149 - 29.510 |
| DSC2 | 18 | 51,250,991 | 2.6 x 10^-2^ | 4,7 | 4.8 x 10^-2^ | 1.009 - 26.529 |
| DDX10 | 14 | 104,762,896 | 2.9 x 10^-2^ | 3,6 | NS | 0.822 - 16.921 |
| LOC102135826 | 13 | 35,499,531 | 3.1 x 10^-2^ | 3,2 | NS | 0.769 - 13.814 |
| LOC102136929 | 1 | 93,120,108 | 3.1 x 10^-2^ | 3,4 | NS | 0.798 - 14.849 |
| LOC102137018 | 1 | 93,151,566 | 3.1 x 10^-2^ | 3,4 | NS | 0.798 - 14.849 |
| LOC102137787 | 1 | 93,241,271 | 3.1 x 10^-2^ | 3,4 | NS | 0.798 - 14.849 |
| LOC102137787 | 1 | 93,241,928 | 3.1 x 10^-2^ | 3,4 | NS | 0.798 - 14.849 |
| MOGS | 13 | 35,448,396 | 3.1 x 10^-2^ | 3,2 | NS | 0.769 - 13.814 |
| LOC101867090 | 3 | 88,848,526 | 3.1 x 10^-2^ | 3,4 | NS | 0.798 - 14.849 |
| SPTA1 | 1 | 93,054,081 | 3.1 x 10^-2^ | 3,4 | NS | 0.798 - 14.849 |
| ZHX3 | 10 | 72,839,745 | 3.1 x 10^-2^ | 3,4 | NS | 0.798 - 14.849 |
| KCNK1 | 1 | 15,898,215 | 3.1 x 10^-2^ | 4,0 | NS | 0.935 - 17.506 |
| ATP10B | 6 | 160,063,849 | 4.3 x 10^-2^ | 3,9 | NS | 0.748 - 26.219 |
| C16H17orf51 | 16 | 21,186,488 | 4.3 x 10^-2^ | 3,6 | NS | 0.467 - 43.083 |
| FETUB | 2 | 91,411,251 | 4.3 x 10^-2^ | 5,2 | 4.0 x 10^-2^ | 0.972 - 34.688 |
| SEL1L2 | 10 | 48,604,843 | 4.3 x 10^-2^ | 3,9 | NS | 0.910 - 31.239 |
| SLC36A3 | 6 | 150,583,542 | 4.7 x 10^-2^ | 3,1 | NS | 0.575 - 18.243 |
| LOC101926846 | X | 78,112,429 | 4.8 x 10^-2^ | 9,0 | 4.5 x 10^-3^ | 1.650 - 89.890 |
| GIPC3 | 19 | 3,561,656 | 4.9 x 10^-2^ | 6,7 | 3.5 x 10^-2^ | 0.860 - Inf |

*location on chromosome (*Macaca fascicularis* RefSeq assembly GCF_000364345.1)

**Supplementary table S4:** LOC102128847-like sequences associated to MHC class I genes of *Mafa* or *Mamu* in databanks.

| Sequences containing MHC genes and a LOC102128847-like sequence | | |
| --- | --- | --- |
| *Mafa (Macaca fascicularis)* |  | *Mamu (Macaca mulatta)* |
| KJ913085; KJ913100; KJ913104; KJ913137; KT330809; KT330903; KT331084; KT331163 |  | AB128049; AC148678; AC148699; AC148713; AC148715; AC210648; KJ456779; KJ456791; KJ456806; KJ456808; KJ456809; KJ456865; KJ456900; KJ456920; KJ456972; KJ456978; KJ457000; KJ457088; KJ457089; KJ457097; KJ457101; KJ457115; KJ489638; KJ489642; KJ489646; KJ489670; KJ489750; KJ489784; KJ489828; KJ489858; KJ489859; KJ913273; KJ913375; KJ913570; KJ913588; KT329542; KT331329; KT331397; KT332412; KT332413; KT332486; KT332522; KT332529; KT332914; KT332925 |

**Supplementary figure S1a**

Legend to Figure S1a : As indicated in the main text of the article, the study of genomic variants in the nine animals selected among the 42 animals of known PVL values at the set point (three with the highest, three with the intermediate, or three with the lowest PVL), polymorphisms segregating exclusively in one of the extreme viral load groups were selected (3 animals with the lowest PVL and 3 animals with the highest PVL), allowing for observations of the disruptive allele in the intermediate group in the intermediate group (3 animals). This led us to characterize 21,664 SNPs potentially associated with the PVL (see Methods for details). The Figure depicts the density of SNPs divided by the total number of SNPs in the window (100 kb). A total of 129 coding genes were identified in the regions of highest densities (above the 95th percentile). Out of these 129 genes only three plausible candidate genes were identified involved in the apoptosis pathway and SIV-infection (FADD : Fas associated via death domain, CASP7 : caspase 7 and MAP3K14:mitogen-activated protein kinase kinase kinase 14).

**Supplementary figure S1b**

Legend to Figure S1b : Following the strategy used by Ericsen and col. ^61^ variant sites were selected for which the animals of the two groups with extreme PVL values (three animals with the highest and three animals with the lowest PVL values) were homozygous but for which the two groups differed (one group was homozygous for the reference base the other was homozygous for the variant base). With this more restrictive approach, a total of 4.532 of such sites were identified dispersed throughout the genome. The numbers of strictly different sites between groups divided by the total number of polymorphic sites in each group were plotted. These highest density regions (above the 95^th^ percentile) differed from the candidates regions characterized by Ericsen et al. 2014 ^61^ (see supplementary note S1). In the highest density regions 120 coding genes were identified of which 17 were found to be related with the SIV infection or the immune response. A description of these 17 genes is given below. Each region (chromosomal locations are in underlined characters) is followed by the brief description of the gene.

chr1:206400000-206500000

**HSPG2 (Heparan Sulfate Proteoglycan 2)**

The HSPG2 gene encodes a cell surface heparan sulfate proteoglycan which mediates the internalization of Tat protein ^62^.

chr1:206600000-206700000

**USP48 : (Ubiquitin Specific Peptidase 48)**

Among its related pathways are Deubiquitination and Ubiquitin-Proteasome Dependent Proteolysis.

This encoded protein may be involved in the regulation of NF-kappa-B activation by TNF receptor superfamily via its interactions with RELA and TRAF2 ^63^.

chr10:39200000-39300000

**CD93 (C1qR : Complement Component 1 Q Subcomponent Receptor 1)**

CD93 is a receptor expressed during early B-cell development. It is reinduced during plasma-cell differentiation and is required for maintenance of antibody secretion and persistence of plasma cells in the bone marrow niche ^64^.

chr10:39200000-39300000

**THBD (Thrombomodulin, CD141 Antigen)**

Recombinant thrombomodulin inhibits lipopolysaccharide-induced inflammatory response by blocking the functions of CD14 ^65^. Thrombomodulin plays a role in inflammation ^66^.

Chr14: 13200000-13400000

**CD6**

CD6 is a cell adhesion molecule that mediates cell-cell contacts and regulates T-cell responses via its interaction with ALCAM/CD166. Contributes to signaling cascades triggered by activation of the TCR/CD3 complex ^67^. Functions as costimulatory molecule; promotes T-cell activation and proliferation. CD6 contributes to the formation and maturation of the immunological synapse. Itactivates the inflammatory response and the secretion of pro-inflammatory cytokines in response to LPS.

Chr18:39900000-40000000

**PIK3C3 (Phosphatidylinositol 3-Kinase Catalytic Subunit Type 3)**

PIK3C3 encoded protein is related to Activated TLR4 signaling. Genetic variation in the promoter region of the PIK3C3 gene is associated with systemic lupus erythematosus ^68^.

Chr20:57800000-57900000

**NFAT5 (Nuclear Factor Of Activated T-Cells 5)**

The product of this gene is a member of the nuclear factors of activated T cells family of transcription factors ^69^. Proteins belonging to this family play a central role in inducible gene transcription during the immune response ^70^.

Chr4:15500000-15900000

**FYN**

FYN encoded protein participates in the downstream signaling which follows T-cell receptor (TCR) stimulation and leads to T-cell differentiation and proliferation ^71^.

Chr4:135100000-135200000

**FKBP5**

FKBP5 is a member of the immunophilin protein family, which play a role in immunoregulation and basic cellular processes involving protein folding and trafficking. This encoded protein is a cis-trans prolyl isomerase that binds to the immunosuppressants FK506 and rapamycin. Regulates the accumulation of glucocorticoid receptor in lymphocytes ^72^.

Chr7:31900000-32400000

**RAB27A**

The encoded protein is member of RAS Oncogene Family. It plays a role in degranulation of NK cells. Mutations in this gene are associated with a severe immunodeficiency (Griscelli syndrome type 2: OMIM 603868).

Chr7:99000000-99100000

**PPP2R3C (Serine/Threonine-Protein Phosphatase 2A Regulatory Subunit B)**

The PPP2R3C encoded protein is a component of a Serine/Threonine-Protein Phosphatase. Homozygous PPP2R3C KO mice exhibit impaired proliferation of B cells. This suggests that it plays a role in B-cell maturation and survival. A variant is associated with the risk to develop atopic dermatitis ^73^.

chrX:35500000-36000000

**CYBB (Superoxide-Generating NADPH Oxidase Heavy Chain Subunit).**

CYBB plays a crucial role in the production of free radicals in phagocytic cells. Its deficiency provokes severe immunodeficiency (OMIM: 300481)

chrX:52600000-52700000

**HUWE1**

The ubiquitin ligase Huwe1 regulates the maintenance and lymphoid commitment of hematopoietic stem cells ^74^.

chrX:87100000-87200000

**USP12 (Ubiquitin Specific Peptidase 12)**

USP12 encoded protein stabilizes the T-cell receptor complex at the cell surface during signaling ^75^.

chrX:87200000-87300000

**RNF19b** (natural killer lytic-associated molecule, NKLAM) is a cytolysis-associated transmembrane protein expressed in natural killer (NK) cells and T lymphocytes following cytokine stimulation ^76^.

chrX:101700000-101800000

**IL1RAPL2**

The protein encoded by this gene is a member of the interleukin 1 receptor family ^77^.

chrX:103900000-104000000

**RNF128**

The protein encoded by this gene is a type I transmembrane which contains a RING zinc-finger motif and has been shown to possess E3 ubiquitin ligase activity. Induced expression of this gene was observed in anergic CD4(+) T cells, which suggested a role in the induction of anergic phenotype Expression of RNF128 limits the activation-induced IL-2 and IL-4 secretion by T lymphocytes ^78^.

**Supplementary note S1:**

As indicated in the legend of figure S1b, the highest density regions (above the 95^th^ percentile) we characterized differed from the candidates regions reported by Ericsen et al. 2014 (see the note 1 below). ^61^ We list below three main factors that could explain the incongruence between studies:

(i) The sample size. The low sample sizes in both studies can produce spurious signals between controllers and progressors due to shared ancestry or even “random” cosegregation of alleles.

(ii) Selection of the animals for the detection of associated variants. Our procedure to select animals for whole-genome sequencing was solely based on viral load 100 days after the virus inoculation, while Ericsen and colleagues not only used viral progression as criterion, but selected individuals sharing the MHC M1 haplotype, which was shown to be enriched in macaques that control SIV progression ^79^. Although their strategy was very powerful to identify SIV control associated variants outside the MHC, the analysis of a cohort of individuals carrying the protective MHC M1 haplotype, potentially shifted the signals of genetic differentiation between controllers and progressors to loci distorting the protective effect of M1.

(iii) Relatedness in the Mauritian macaque population. All present-day cynomolgus macaques in Mauritius descend from a founder population of around 20 individuals deposited in the island 500 years ago ^80,81^. The use of isolated populations has a long history in genetic mapping, with benefits including reduced genetic diversity and reduced environmental heterogeneity ^82^. Nevertheless, studies based on inbred cohorts introduce analytic challenges, as extensive relatedness between subjects violates the assumptions of independence upon which traditional association studies are based. In case of Mauritius macaque population, it is certainly very difficult to exclude the possibility that some of the highly differentiated segments of the genome are due to cryptic relatedness between individuals. Such scenario could explain the differences between studies, as sampling different lineages or groups of related individuals would yield different sets of highly differentiated loci.

**Supplementary figure S2. Locations of the RIMKLB gene and pseudogenes in the *Macaca fascicularis* genome.**

**Legend of the supplementary figure 2:**

To refine the genotype of the MHC LOC102128847-like locus, we extracted a set of seven reference sequences (three of *Mafa* and four of *Mamu*) of 201 base pairs (100 bp 5’ and 100 bp 3’ to the polymorphic site “G/A”) from databanks. The mapping of the sequence reads and reference sequences were performed by the GS Reference Mapper Ver. 3.0 software (Roche). The mapping parameter was set to 100% and 99% of matched between the sequence reads and the reference sequences to avoid mis-mapping of the sequence reads. Then we excluded the sequences corresponding to the functional RIMKLB gene (chromosome 11) and the two retro-pseudogenes on chromosomes 1 and 17.

The sequences characterized in the 201 bp mapping region are described by reference to the LOC102128847 sequences of the *Macaca fascicularis* 5.0 whole genome shotgun sequence. The presence of LOC102128847 and/or LOC102128847NEW was detected in all 42 animals under study and most probably corresponded to two alleles of the chromosome 14 RIMKLB pseudogene. We also identified the presence of sequences corresponding to MHC-associated LOC102128847-like locus (KT330809-new, KJ48959, KJ913137, KT331084, KT330809, in one, five, 12, 18 and 17 animals, respectively).

The LOC102128847-like alleles associated with MHC haplotypes M2 and M4, had a “A” a the SNP site, while LOC102128847-like alleles associated with MHC haplotypes M3, M6 and M7 were had a “G” at the SNP site. Finally, we did not find LOC102128847-like associated with MHC haplotypes M1 and M5. The table inside the figure gives for each LOC102128847-like allele the reference sequence which was characterized in a region of 201 nucleotides centered around the SNP site.

**References of supplementary tables and figures**.

1 Cingolani, P. *et al.* A program for annotating and predicting the effects of single nucleotide polymorphisms, SnpEff: SNPs in the genome of Drosophila melanogaster strain w1118; iso-2; iso-3. *Fly* **6**, 80-92, doi:10.4161/fly.19695 (2012).

2 Brass, A. L. *et al.* Identification of host proteins required for HIV infection through a functional genomic screen. *Science* **319**, 921-926, doi:10.1126/science.1152725 (2008).

3 Williams, C. A., Abbink, T. E., Jeang, K. T. & Lever, A. M. Identification of RNA helicases in human immunodeficiency virus 1 (HIV-1) replication - a targeted small interfering RNA library screen using pseudotyped and WT HIV-1. *The Journal of general virology* **96**, 1484-1489, doi:10.1099/vir.0.000092 (2015).

4 Konig, R. *et al.* Global analysis of host-pathogen interactions that regulate early-stage HIV-1 replication. *Cell* **135**, 49-60, doi:10.1016/j.cell.2008.07.032 (2008).

5 Yeung, M. L., Houzet, L., Yedavalli, V. S. & Jeang, K. T. A genome-wide short hairpin RNA screening of jurkat T-cells for human proteins contributing to productive HIV-1 replication. *The Journal of biological chemistry* **284**, 19463-19473, doi:10.1074/jbc.M109.010033 (2009).

6 Liu, L. *et al.* A whole genome screen for HIV restriction factors. *Retrovirology* **8**, 94, doi:10.1186/1742-4690-8-94 (2011).

7 De, S. K., Venkateshan, C. N., Seth, P., Gajdusek, D. C. & Gibbs, C. J. Adenovirus-mediated human immunodeficiency virus-1 Nef expression in human monocytes/macrophages and effect of Nef on downmodulation of Fcgamma receptors and expression of monokines. *Blood* **91**, 2108-2117 (1998).

8 Mrowiec, T., Melchar, C. & Gorski, A. HIV-protein-mediated alterations in T cell interactions with the extracellular matrix proteins and endothelium. *Archivum immunologiae et therapiae experimentalis* **45**, 255-259 (1997).

9 Lopez-Huertas, M. R. *et al.* The presence of HIV-1 Tat protein second exon delays fas protein-mediated apoptosis in CD4+ T lymphocytes: a potential mechanism for persistent viral production. *The Journal of biological chemistry* **288**, 7626-7644, doi:10.1074/jbc.M112.408294 (2013).

10 Kim, J. O. *et al.* Lysis of human immunodeficiency virus type 1 by a specific secreted human phospholipase A2. *Journal of virology* **81**, 1444-1450, doi:10.1128/JVI.01790-06 (2007).

11 Parker, Z. F. *et al.* Platelet Factor 4 Inhibits and Enhances HIV-1 Infection in a Concentration-Dependent Manner by Modulating Viral Attachment. *AIDS research and human retroviruses* **32**, 705-717, doi:10.1089/AID.2015.0344 (2016).

12 Jager, S. *et al.* Global landscape of HIV-human protein complexes. *Nature* **481**, 365-370, doi:10.1038/nature10719 (2011).

13 Haidar, M., Seddiki, N., Gluckman, J. C. & Gattegno, L. Carbohydrate binding properties of the envelope glycoproteins of human immunodeficiency virus type 1. *Glycoconjugate journal* **9**, 315-323 (1992).

14 Gregoire, I. P., Rabourdin-Combe, C. & Faure, M. Autophagy and RNA virus interactomes reveal IRGM as a common target. *Autophagy* **8**, 1136-1137, doi:10.4161/auto.20339 (2012).

15 Gregoire, I. P. *et al.* IRGM is a common target of RNA viruses that subvert the autophagy network. *PLoS pathogens* **7**, e1002422, doi:10.1371/journal.ppat.1002422 (2011).

16 Milev, M. P., Ravichandran, M., Khan, M. F., Schriemer, D. C. & Mouland, A. J. Characterization of staufen1 ribonucleoproteins by mass spectrometry and biochemical analyses reveal the presence of diverse host proteins associated with human immunodeficiency virus type 1. *Frontiers in microbiology* **3**, 367, doi:10.3389/fmicb.2012.00367 (2012).

17 Gupta, K., Ott, D., Hope, T. J., Siliciano, R. F. & Boeke, J. D. A human nuclear shuttling protein that interacts with human immunodeficiency virus type 1 matrix is packaged into virions. *Journal of virology* **74**, 11811-11824 (2000).

18 Shoeman, R. L. *et al.* Cleavage of human and mouse cytoskeletal and sarcomeric proteins by human immunodeficiency virus type 1 protease. Actin, desmin, myosin, and tropomyosin. *The American journal of pathology* **142**, 221-230 (1993).

19 Shoeman, R. L., Hartig, R., Hauses, C. & Traub, P. Organization of focal adhesion plaques is disrupted by action of the HIV-1 protease. *Cell biology international* **26**, 529-539 (2002).

20 Valente, S. T., Gilmartin, G. M., Mott, C., Falkard, B. & Goff, S. P. Inhibition of HIV-1 replication by eIF3f. *Proceedings of the National Academy of Sciences of the United States of America* **106**, 4071-4078, doi:10.1073/pnas.0900557106 (2009).

21 Naji, S. *et al.* Host cell interactome of HIV-1 Rev includes RNA helicases involved in multiple facets of virus production. *Molecular & cellular proteomics : MCP* **11**, M111 015313, doi:10.1074/mcp.M111.015313 (2012).

22 Li, X. Y. & Green, M. R. The HIV-1 Tat cellular coactivator Tat-SF1 is a general transcription elongation factor. *Genes & development* **12**, 2992-2996 (1998).

23 Herrera, R. *et al.* Human beta-defensins 2 and -3 cointernalize with human immunodeficiency virus via heparan sulfate proteoglycans and reduce infectivity of intracellular virions in tonsil epithelial cells. *Virology* **487**, 172-187, doi:10.1016/j.virol.2015.09.025 (2016).

24 Woollard, S. M., Bhargavan, B., Yu, F. & Kanmogne, G. D. Differential effects of Tat proteins derived from HIV-1 subtypes B and recombinant CRF02_AG on human brain microvascular endothelial cells: implications for blood-brain barrier dysfunction. *Journal of cerebral blood flow and metabolism : official journal of the International Society of Cerebral Blood Flow and Metabolism* **34**, 1047-1059, doi:10.1038/jcbfm.2014.54 (2014).

25 Johnson, T. P. *et al.* Induction of IL-17 and nonclassical T-cell activation by HIV-Tat protein. *Proceedings of the National Academy of Sciences of the United States of America* **110**, 13588-13593, doi:10.1073/pnas.1308673110 (2013).

26 Park, S. E. *et al.* Expression profiles and pathway analysis in HEK 293 T cells overexpressing HIV-1 Tat and nucleocapsid using cDNA microarray. *Journal of microbiology and biotechnology* **17**, 154-161 (2007).

27 Madani, N. *et al.* Implication of the lymphocyte-specific nuclear body protein Sp140 in an innate response to human immunodeficiency virus type 1. *Journal of virology* **76**, 11133-11138 (2002).

28 Harman, A. N. *et al.* HIV-1-infected dendritic cells show 2 phases of gene expression changes, with lysosomal enzyme activity decreased during the second phase. *Blood* **114**, 85-94, doi:10.1182/blood-2008-12-194845 (2009).

29 Luo, M. *et al.* A genetic polymorphism of FREM1 is associated with resistance against HIV infection in the Pumwani sex worker cohort. *Journal of virology* **86**, 11899-11905, doi:10.1128/JVI.01499-12 (2012).

30 Anand, A. R., Zhao, H., Nagaraja, T., Robinson, L. A. & Ganju, R. K. N-terminal Slit2 inhibits HIV-1 replication by regulating the actin cytoskeleton. *Retrovirology* **10**, 2, doi:10.1186/1742-4690-10-2 (2013).

31 Fukuhara, T. *et al.* Utilization of host SR protein kinases and RNA-splicing machinery during viral replication. *Proceedings of the National Academy of Sciences of the United States of America* **103**, 11329-11333, doi:10.1073/pnas.0604616103 (2006).

32 Marras, F. *et al.* Natural killer cells in HIV controller patients express an activated effector phenotype and do not up-regulate NKp44 on IL-2 stimulation. *Proceedings of the National Academy of Sciences of the United States of America* **110**, 11970-11975, doi:10.1073/pnas.1302090110 (2013).

33 Yang, W., Jackson, B. & Zhang, H. Identification of glycoproteins associated with HIV latently infected cells using quantitative glycoproteomics. *Proteomics* **16**, 1872-1880, doi:10.1002/pmic.201500215 (2016).

34 Kurosaki, T. & Tsukada, S. BLNK: connecting Syk and Btk to calcium signals. *Immunity* **12**, 1-5 (2000).

35 Connolly, D. J. & Bowie, A. G. The emerging role of human PYHIN proteins in innate immunity: implications for health and disease. *Biochemical pharmacology* **92**, 405-414, doi:10.1016/j.bcp.2014.08.031 (2014).

36 Hoye, A. M., Couchman, J. R., Wewer, U. M., Fukami, K. & Yoneda, A. The newcomer in the integrin family: integrin alpha9 in biology and cancer. *Advances in biological regulation* **52**, 326-339, doi:10.1016/j.jbior.2012.03.004 (2012).

37 Rempel, H. *et al.* Monocyte activation in HIV/HCV coinfection correlates with cognitive impairment. *PloS one* **8**, e55776, doi:10.1371/journal.pone.0055776 (2013).

38 Gateva, V. *et al.* A large-scale replication study identifies TNIP1, PRDM1, JAZF1, UHRF1BP1 and IL10 as risk loci for systemic lupus erythematosus. *Nature genetics* **41**, 1228-1233, doi:10.1038/ng.468 (2009).

39 Ishibashi, K., Suzuki, M., Sasaki, S. & Imai, M. Identification of a new multigene four-transmembrane family (MS4A) related to CD20, HTm4 and beta subunit of the high-affinity IgE receptor. *Gene* **264**, 87-93 (2001).

40 Dai, K. Z. *et al.* The T cell regulator gene SH2D2A contributes to the genetic susceptibility of multiple sclerosis. *Genes and immunity* **2**, 263-268, doi:10.1038/sj.gene.6363774 (2001).

41 Martin, M. P. & Carrington, M. Immunogenetics of HIV disease. *Immunological reviews* **254**, 245-264, doi:10.1111/imr.12071 (2013).

42 Coulon, P. G. *et al.* HIV-Infected Dendritic Cells Present Endogenous MHC Class II-Restricted Antigens to HIV-Specific CD4+ T Cells. *J Immunol* **197**, 517-532, doi:10.4049/jimmunol.1600286 (2016).

43 Sauermann, U. *et al.* Homozygosity for a conserved Mhc class II DQ-DRB haplotype is associated with rapid disease progression in simian immunodeficiency virus-infected macaques: results from a prospective study. *The Journal of infectious diseases* **182**, 716-724, doi:10.1086/315800 (2000).

44 Giraldo-Vela, J. P. *et al.* The major histocompatibility complex class II alleles Mamu-DRB1*1003 and -DRB1*0306 are enriched in a cohort of simian immunodeficiency virus-infected rhesus macaque elite controllers. *Journal of virology* **82**, 859-870, doi:10.1128/JVI.01816-07 (2008).

45 Eggensperger, S. & Tampe, R. The transporter associated with antigen processing: a key player in adaptive immunity. *Biological chemistry* **396**, 1059-1072, doi:10.1515/hsz-2014-0320 (2015).

46 Lankat-Buttgereit, B. & Tampe, R. The transporter associated with antigen processing: function and implications in human diseases. *Physiological reviews* **82**, 187-204, doi:10.1152/physrev.00025.2001 (2002).

47 Guergnon, J. *et al.* Single-Nucleotide Polymorphism-Defined Class I and Class III Major Histocompatibility Complex Genetic Subregions Contribute to Natural Long-term Nonprogression in HIV Infection. *Journal of Infectious Diseases* **205**, 718-724, doi:10.1093/infdis/jir833 (2012).

48 Pereyra, F. P. *et al.* The Major Genetic Determinants of HIV-1 Control Affect HLA Class I Peptide Presentation. *Science* **330**, 1551-1557, doi:10.1126/science.1195271 (2010).

49 Goulder, P. J. R. & Walker, B. D. HIV and HLA Class I: An Evolving Relationship. *Immunity* **37**, 426-440, doi:10.1016/j.immuni.2012.09.005 (2012).

50 Mann, D. L. *et al.* Major histocompatibility complex genotype is associated with disease progression and virus load levels in a cohort of human immunodeficiency virus type 1-infected Caucasians and African Americans. *The Journal of infectious diseases* **178**, 1799-1802 (1998).

51 Liu, C. L. *et al.* Association of polymorphisms in human leukocyte antigen class I and transporter associated with antigen processing genes with resistance to human immunodeficiency virus type 1 infection. *Journal of Infectious Diseases* **187**, 1404-1410, doi:Doi 10.1086/374394 (2003).

52 Silver, Z. A. & Watkins, D. I. The role of MHC class I gene products in SIV infection of macaques. *Immunogenetics* **69**, 511-519, doi:10.1007/s00251-017-0997-3 (2017).

53 Aarnink, A. *et al.* Influence of the MHC genotype on the progression of experimental SIV infection in the Mauritian cynomolgus macaque. *Immunogenetics* **63**, 267-274, doi:10.1007/s00251-010-0504-6 (2011).

54 Mee, E. T. *et al.* Mhc haplotype H6 is associated with sustained control of SIVmac251 infection in Mauritian cynomolgus macaques. *Immunogenetics* **61**, 327-339, doi:10.1007/s00251-009-0369-8 (2009).

55 Antony, J. M. & MacDonald, K. S. A critical analysis of the cynomolgus macaque, Macaca fascicularis, as a model to test HIV-1/SIV vaccine efficacy. *Vaccine* **33**, 3073-3083, doi:10.1016/j.vaccine.2014.12.004 (2015).

56 Burwitz, B. J. *et al.* Mauritian cynomolgus macaques share two exceptionally common major histocompatibility complex class I alleles that restrict simian immunodeficiency virus-specific CD8+ T cells. *Journal of virology* **83**, 6011-6019, doi:10.1128/JVI.00199-09 (2009).

57 Sauermann, U. *et al.* Mhc class I haplotypes associated with survival time in simian immunodeficiency virus (SIV)-infected rhesus macaques. *Genes and immunity* **9**, 69-80, doi:10.1038/sj.gene.6364448 (2008).

58 Nomura, T. & Matano, T. Association of MHC-I genotypes with disease progression in HIV/SIV infections. *Frontiers in microbiology* **3**, 234, doi:10.3389/fmicb.2012.00234 (2012).

59 Loffredo, J. T. *et al.* Mamu-B*08-positive macaques control simian immunodeficiency virus replication. *Journal of virology* **81**, 8827-8832, doi:10.1128/JVI.00895-07 (2007).

60 Muhl, T., Krawczak, M., Ten Haaft, P., Hunsmann, G. & Sauermann, U. MHC class I alleles influence set-point viral load and survival time in simian immunodeficiency virus-infected rhesus monkeys. *J Immunol* **169**, 3438-3446 (2002).

61 Ericsen, A. J. *et al.* Whole genome sequencing of SIV-infected macaques identifies candidate loci that may contribute to host control of virus replication. *Genome Biol* **15**, 478, doi:10.1186/s13059-014-0478-z (2014).

62 Argyris, E. G. *et al.* The perlecan heparan sulfate proteoglycan mediates cellular uptake of HIV-1 Tat through a pathway responsible for biological activity. *Virology* **330**, 481-486, doi:10.1016/j.virol.2004.10.011 (2004).

63 Zhang, X. *et al.* An Interaction Landscape of Ubiquitin Signaling. *Molecular cell* **65**, 941-955 e948, doi:10.1016/j.molcel.2017.01.004 (2017).

64 Chevrier, S. *et al.* CD93 is required for maintenance of antibody secretion and persistence of plasma cells in the bone marrow niche. *Proceedings of the National Academy of Sciences of the United States of America* **106**, 3895-3900, doi:10.1073/pnas.0809736106 (2009).

65 Ma, C. Y. *et al.* Recombinant Thrombomodulin Inhibits Lipopolysaccharide-Induced Inflammatory Response by Blocking the Functions of CD14. *Journal of Immunology* **194**, 1905-1915, doi:10.4049/jimmunol.1400923 (2015).

66 Conway, E. M. Thrombomodulin and its role in inflammation. *Semin Immunopathol* **34**, 107-125, doi:10.1007/s00281-011-0282-8 (2012).

67 Santos, R. F., Oliveira, L. & Carmo, A. M. Tuning T Cell Activation: The Function of CD6 At the Immunological Synapse and in T Cell Responses. *Curr Drug Targets* **17**, 630-639, doi:10.2174/1389450116666150531152439 (2016).

68 Kariuki, S. N. et al. Promoter Variant of PIK3C3 Is Associated with Autoimmunity against Ro and Sm Epitopes in African-American Lupus Patients. *J Biomed Biotechnol* **2010**, Article ID 826434, 7 pages, doi.org/10.1155/2010/826434 (2010)

69 Berga-Bolanos, R., Drews-Elger, K., Aramburu, J. & Lopez-Rodriguez, C. NFAT5 regulates T lymphocyte homeostasis and CD24-dependent T cell expansion under pathologic hypernatremia. *J Immunol* **185**, 6624-6635, doi:10.4049/jimmunol.1001232 (2010).

70 Tellechea, M., Buxade, M., Tejedor, S., Aramburu, J. & Lopez-Rodriguez, C. NFAT5-Regulated Macrophage Polarization Supports the Proinflammatory Function of Macrophages and T Lymphocytes. *Journal of Immunology* **200**, 305-315, doi:10.4049/jimmunol.1601942 (2018).

71 Palacios, E. H. & Weiss, A. Function of the Src-family kinases, Lck and Fyn, in T-cell development and activation. *Oncogene* **23**, 7990-8000, doi:10.1038/sj.onc.1208074 (2004).

72 Lukic, I. *et al.* Accumulation of Cytoplasmic Glucocorticoid Receptor Is Related to Elevation of FKBP5 in Lymphocytes of Depressed Patients. *J Mol Neurosci* **55**, 951-958, doi:10.1007/s12031-014-0451-z (2015).

73 Paternoster, L. *et al.* Multi-ancestry genome-wide association study of 21,000 cases and 95,000 controls identifies new risk loci for atopic dermatitis. *Nature genetics* **47**, 1449-+, doi:10.1038/ng.3424 (2015).

74 King, B. *et al.* The ubiquitin ligase Huwe1 regulates the maintenance and lymphoid commitment of hematopoietic stem cells. *Nat Immunol* **17**, 1312-1321, doi:10.1038/ni.3559 (2016).

75 Jahan, A. S. *et al.* Usp12 stabilizes the T-cell receptor complex at the cell surface during signaling. *Proceedings of the National Academy of Sciences of the United States of America* **113**, E705-E714, doi:10.1073/pnas.1521763113 (2016).

76 Kozlowski, M., Schorey, J., Portis, T., Grigoriev, V. & Kornbluth, J. NK lytic-associated molecule: A novel gene selectively expressed in cells with cytolytic function. *Journal of Immunology* **163**, 1775-1785 (1999).

77 Sana, T. R., Debets, R., Timans, J. C., Bazan, J. F. & Kastelein, R. A. Computational identification, cloning, and characterization of IL-1R9, a novel interleukin-1 receptor-like gene encoded over an unusually large interval of human chromosome Xq22.2-q22.3. *Genomics* **69**, 252-262, doi:10.1006/geno.2000.6328 (2000).

78 Anandasabapathy, N. *et al.* GRAIL: An E3 ubiquitin ligase that inhibits cytokine gene transcription is expressed in anergic CD4(+) T cells. *Immunity* **18**, 535-547, doi:Doi 10.1016/S1074-7613(03)00084-0 (2003).

79 Budde, M. L. *et al.* Specific CD8(+) T Cell Responses Correlate with Control of Simian Immunodeficiency Virus Replication in Mauritian Cynomolgus Macaques. *Journal of virology* **86**, 7596-7604, doi:10.1128/Jvi.00716-12 (2012).

80 Osada, N., Hettiarachchi, N., Babarinde, I. A., Saitou, N. & Blancher, A. Whole-Genome Sequencing of Six Mauritian Cynomolgus Macaques (Macaca fascicularis) Reveals a Genome-Wide Pattern of Polymorphisms under Extreme Population Bottleneck. *Genome Biol Evol* **7**, 821-830, doi:10.1093/gbe/evv033 (2015).

81 Sussman, R. W. & Tattersall, I. Distribution, Abundance, and Putative Ecological Strategy of Macaca-Fascicularis on the Island of Mauritius, Southwestern Indian-Ocean. *Folia Primatol* **46**, 28-43, doi:Doi 10.1159/000156234 (1986).

82 Lowe, J. K. *et al.* Genome-Wide Association Studies in an Isolated Founder Population from the Pacific Island of Kosrae. *Plos Genet* **5**, e1000365. doi.org/10.1371/journal.pgen.1000365 (2009).
